# Internal fibrinolysis of fibrin clots is driven by pore expansion

**DOI:** 10.1038/s41598-024-52844-4

**Published:** 2024-02-01

**Authors:** Rebecca A. Risman, Bradley Paynter, Victoria Percoco, Mitali Shroff, Brittany E. Bannish, Valerie Tutwiler

**Affiliations:** 1 Department of Biomedical Engineering, Rutgers University, 599 Taylor Road, Piscataway, NJ 08854 USA; 2 Department of Mathematics and Statistics, University of Central Oklahoma, Edmond, USA; 3 Department of Cell Biology and Neuroscience, Rutgers University, Piscataway, USA

**Keywords:** Computational models, Experimental models of disease, Embolism, Thromboembolism, Thrombosis, Biomedical engineering, Applied mathematics, Biological physics, Confocal microscopy, Spectrophotometry, Computational biology and bioinformatics

## Abstract

Blood clots, which are composed of blood cells and a stabilizing mesh of fibrin fibers, are critical in cessation of bleeding following injury. However, their action is transient and after performing their physiological function they must be resolved through a process known as fibrinolysis. Internal fibrinolysis is the degradation of fibrin by the endogenous or innate presence of lytic enzymes in the bloodstream; under healthy conditions, this process regulates hemostasis and prevents bleeding or clotting. Fibrin-bound tissue plasminogen activator (tPA) converts nearby plasminogen into active plasmin, which is bound to the fibrin network, breaking it down into fibrin degradation products and releasing the entrapped blood cells. It is poorly understood how changes in the fibrin structure and lytic protein ratios influence the biochemical regulation and behavior of internal fibrinolysis. We used turbidity kinetic tracking and microscopy paired with mathematical modeling to study fibrin structure and lytic protein ratios that restrict internal fibrinolysis. Analysis of simulations and experiments indicate that fibrinolysis is driven by pore expansion of the fibrin network. We show that this effect is strongly influenced by the ratio of fibrin:tPAwhen compared to absolute tPA concentration. Thus, it is essential to consider relative protein concentrations when studying internal fibrinolysis both experimentally and in the clinic. An improved understanding of effective internal lysis can aid in development of better therapeutics for the treatment of bleeding and thrombosis.

## Introduction

Hemostasis, or the formation of a stable blood clot, is the balance of coagulation (clot formation) and fibrinolysis (clot degradation) to prevent bleeding and maintain a healthy vessel for steady blood flow to transport necessary nutrients to organs^1–8^. Elevation or depletion of coagulation factors, such as fibrin(ogen) or thrombin, as a result of disease or injury can lead to severe complications^9,10^. Fibrin(ogen), which is the main structural component of a blood clot, polymerizes into a fibrin network that encases the cellular components of the blood and aids in wound healing^11–13^. Blood clotting is initiated when an injury to the blood vessel occurs, activating platelets and converting prothrombin into thrombin (Factor II in the coagulation cascade). Thrombin cleaves the fibrinopeptides on fibrinogen to create a fibrin monomer with available cross-linking sites for protofibril formation. This cycle continues in the process of lateral aggregation and packing to create fibrin fibers and finally the formation of a polymerized fibrin network^11–13^. Key structural differences between clots created under various conditions include fibrin network density, pore size, fibrin fiber diameter, and number of fibers per region. Changes in fiber diameter result from lateral aggregation of fibers. If the blood clot forms slowly, as in the case of lower thrombin concentration, the fibers have more time to laterally aggregate, creating thicker, longer fibers^14,15^. On the other hand, elevation of fibrinogen leads to thicker fibers and denser networks^16^. Pores, or holes, in the fibrin network develop as individual fibers intertwine with each other to form a mesh; these are the regions in which lytic agents can diffuse or perfuse through the clot during degradation. These pores are present not only in fibrin clots but also in clinical thrombi and clots made with whole blood^17^. Structural changes in the fibrin network alter a clot’s susceptibility or resistance to clot degradation^9,17–19^.

Fibrinolysis is initiated when a plasminogen activator, commonly tissue plasminogen activator (tPA), converts plasminogen into plasmin. Plasmin subsequently binds to fibrin and transversely cuts the fiber via enzymatic degradation^20^. tPA molecules naturally flow in the bloodstream at 5 ng/mL concentration to prevent the formation of an undesired clot and maintain hemostasis^12^. It has been thought that tPA levels can be elevated through injury, initiation of clotting cascade, endothelial activation, exercise, and disease^21^. Furthermore, altered tPA concentration is thought to be a marker for risk of myocardial infarction^22^. Internal lysis occurs when tPA molecules are trapped in the clot while it is forming and initiate the degradation of fibrin from within the clot. The structural changes during clot breakdown are currently unknown.

When a clot cannot be degraded by internal lysis alone, such as in the event of heart attack or stroke, tPA is exogenously delivered in a process termed external fibrinolysis^23^. Modeling simulations of external fibrinolysis, supplemented with experimentation, revealed that the ratio between tPA and fibrin molecules (rather than the overall concentration of tPA present) plays a paramount role in the efficiency of clot breakdown^24,25^. The composition of the clot may inform about the tPA drug dosage for patients to prevent excessive bleeding while also treating the patient within the effective time window^25,26^. However, this finding has only been reported for external lysis and has not yet been studied for internal fibrinolysis. Establishing a better understanding of the role of relative protein concentrations and clot structure on the behavior of internal lysis can inform about ways to improve external lysis and clinical advancements^27^.

Fibrin fiber diameter and network density, along with other clot structure parameters, can either facilitate or hinder the breakdown of the clot^3,24,28–31^. It is currently unknown which of these parameters drives the rate and extent of internal fibrinolysis. We and others have looked at the role of fibrinogen and thrombin concentration on the clot network, but many of these structural changes occur simultaneously. For example, increasing fibrinogen concentration has been shown to increase both fiber diameter and network density^16,32^. Moreover, it has been shown for external fibrinolysis that individually thick fibers lyse slower than individually thin fibers, but a densely packed clot with thin fibers will lyse slower than a loosely packed clot with thick fibers^30^. Some work was performed on internal lysis, looking at the fibrin architecture on one fibrinogen and thrombin concentration^29^. Furthermore, these studies utilized only one technique (microscopy) without probing the polymerization and degradation times and rates^33^.

A fixed concentration of tPA is typically used for internal lysis experiments, despite changes in other protein concentrations, such as fibrinogen or thrombin. These experiments do not accurately resemble clinical conditions in which increased fibrinogen may lead to increased tPA^34^. Moreover, clinicians utilize fibrinogen replacement, either concentrate or cryoprecipitate, to aid in management of hemodilution, but they do not consider endogenous tPA presence^35,36^. Interestingly, fibrinogen is not always a standard measurement to monitor thrombotic risk^37^. For these reasons, varying the concentration of tPA to accurately match the alteration in clotting proteins, such as fibrinogen, would be more representative of physiological conditions. In this work, we study internal fibrinolysis both when there is a fixed ratio of tPA molecules to fibrin molecules and when there is a fixed tPA concentration, which could help understand the regulated balance of clotting factors and lytic agents during hemostasis. Imbalance of these factors leads to medical complications.

Mathematical modeling can vary clot structural changes individually in a way that cannot be done experimentally^25,31,38–41^. Our previous work on external fibrinolysis proposed the need for an optimal tPA to fibrin binding affinity for a novel therapeutic to prevent the tPA molecules from getting stuck on the fibers in the dense periphery of a contracted clot^31^. Furthermore, it has been shown that external fibrinolysis occurs as a lysis front in which the tPA molecules (and hence lysis of fibers) are restricted to a narrow region at the edge of the clot^29,39^ but a similar understanding of the pattern of internal lysis is lacking. How the individual structural changes of a fibrin clot impact the rate, delay in, and pattern of internal fibrinolysis is poorly understood. Here, we build upon our previously published 3-dimensional mathematical model of external fibrinolysis to study internal lysis of fibrin clots. We use laboratory experiments to verify the mathematical predictions about the effect of pore size and demonstrate the heterogeneity of pore expansion that culminates in complete dissolution of the fibrin network.

## Methods

### Experimental conditions

Two sets of experiments were performed to compare fixed concentration of tPA and fixed tPA: fibrin molecule ratio (Table 1). Fixed concentration keeps the amount of tPA constant regardless of fibrinogen concentration; fixed ratio varies the amount of tPA to adjust for changing fibrinogen concentration (i.e., increasing the tPA concentration if the fibrinogen concentration increases). Confocal microscopy images of fibrin clots with the range of fibrinogen concentrations presented here (0.23–2.1 mg/mL) can be seen in the supplement (Supp. Figure 1).

**Table 1:** Experimental design for fixed concentration and fixed ratio for increasing fibrinogen concentration with internal fibrinolysis. Molecular weight (MW) of fibrinogen is 340 kilodaltons; MW of tPA is 68 kilodaltons.

Fibrinogen (mg/mL)	Fixed concentration (FC)		Fixed ratio (FR)	
	tPA (ng/mL)	Ratio (tPA ng/fibrinogen mg)	tPA (ng/mL)	Ratio (tPA ng/fibrinogen mg)
0.23	40	173.9	8	33.3
0.70	40	57.1	24	33.3
1.20	40	33.3	40	33.3
2.10	40	19.0	72	33.3

Commercially available human-pooled blood plasma (Cone Bioproducts #5781) was warmed to 37 °C prior to all experiments. First, a fixed concentration of tPA (40 ng/mL, final concentration) was added to the plasma mix before clotting was initiated. This concentration was chosen as the baseline tPA concentration as it allows the clot to fully form before lysis occurs. In a second set of experiments, a fixed ratio of tPA to fibrin molecules was established. The 1.2 mg/mL fibrinogen sample received 40 ng/mL tPA; 2.1 mg/mL received 1.8 times more tPA (72 ng/mL), 0.70 mg/mL and 0.23 mg/mL received 1.67 and 5- times less tPA (24 and 8 ng/mL), respectively. Therefore, the 1.2 mg/mL samples for both fixed ratio and fixed concentration are the same conditions and behave as the fulcrum for the increase/decrease in tPA as the fibrinogen concentration increases/decreases, respectively. Going forward, we will refer to these conditions as “fixed concentration” or “FC” and “fixed ratio” or “FR”. Table 1 highlights the fibrinogen and tPA conditions established to experimentally fix either the concentration of tPA or the ratio of tPA to fibrin.

#### Turbidity

To analyze the biochemical regulation of clot formation and fibrinolysis, we performed a standard microplate turbidity assay of samples with increasing concentrations of fibrinogen (0.23, 0.70, 1.2, 2.1 mg/mL, final concentration). Concentrations were achieved by diluting plasma with a fibrinogen concentration of 2.9 mg/mL with a buffer solution (50 mM Tris, 70 mM NaCl, 1 mg/mL BSA, starting concentrations), as has been previously described^31,42,43^. To confirm results seen were due to changes in fibrinogen concentration rather than dilution of all plasma factors, we performed a subset of experiments in which the diluent was fibrinogen-depleted plasma. We saw insignificant differences in rate of formation, maximum optical density, time to 50% lysis, and degradation rate when samples were diluted with buffer vs fibrinogen-depleted plasma (Supp. Figure 1). Lysis was initiated with tPA with final concentrations shown in Table 1. tPA was added directly into the plasma mix. An activation mix with thrombin (0.1 U/mL, final concentration), CaCl_2_ (25 mM, final concentration), and buffer (50 mM Tris, 70 mM NaCl, 1 mg/mL BSA, starting concentrations) was used to initiate clotting. Samples were run in triplicate on three distinct days (N = 3 days, n = 9). Samples were tracked for four hours every 15 s on a Molecular Devices SpectraMax at 405 nm.

Data was normalized to the first point to remove background. Next, samples were normalized to the maximum value to view the data as fractions of the clot; this normalization removes bias from natural optical density (OD) changes in plasma due to fibrinogen concentration. Slight discrepancies may arise in the normalized data due to biological and technical variability that manifest in slight differences in the starting OD. Every 9 data points were plotted on Prism from the plots shown (Fig. 2A,B) for easier visualization; plots with and without these data points were compared to ensure no information was lost. All analysis was performed with the full data set.

The maximum optical density, rate of lysis, and time to 50% lysis were measured and recorded. The maximum optical density was recorded as the maximum value when the curves are normalized to the first point. To observe the overall degradation rate, rate to 50% lysis, and rate after 50% lysis, we calculated the slope of the normalized to maximum optical density curves at three distinct degradation ranges (20–80%, 20–50%, and 50–80%, respectively). The time to 50% lysis was recorded as the time when the lysis curves normalized to maximum optical density are closest to 0.5 (fraction of the clot). Lysis lag time was reported as time to 5% degradation (0.95 fraction of clot after clot was fully formed).

#### Timelapse microscopy

Timelapse recordings were used to visually examine the degradation of the fibrin network over time. Clots were formed with the same clot conditions as for turbidity with the addition of a fluorophore: commercially available human-pooled plasma (2.9 mg/mL starting fibrinogen concentration, Cone Bioproducts # 5781) was diluted with buffer (50 mM Tris, 70 mM NaCl, 1 mg/mL BSA, starting concentrations) to achieve the desired fibrinogen concentrations (0.23, 0.70, 1.2, and 2.1 mg/mL), and an appropriate amount of tPA (Table 1) was added. CaCl_2_ (25 mM, final concentration) and thrombin (0.1 U/mL, final concentration) were added immediately prior to addition of the sample to the test well and beginning image collection. Images were captured at three different regions of the clot every 30 s until completion of lysis on a Zeiss 78 confocal microscope (NA = 1.2) at 40 × magnification water objective and 1024 × 1024 pixels (Supp. Figure 10). FIJI (ImageJ) was used to manually measure the pore sizes of every five images to track their growth over time. Specific methods can be found in the supplement (Supp. Figure 11).

Heat maps were created for each fibrinogen concentration at five different time points. The time points chosen were indicative of a fully formed clot, three points during the lysis process at which there were noticeable changes in the structure, and the last time points recorded. Standard deviation of the pore size of each region across each time point was found.

### Modeling

Unlike our previously published models that studied external lysis^25,31,39^, where tPA is introduced as a bolus in a fibrin-free region abutting the clot, here we investigate internal lysis by initializing tPA inside the clot. Because network density (number of fibers per region, or pore size) and fiber diameter (thickness) play key roles in fibrinolysis, the model is run for two different diameters, 72.7 nm (“thin” fiber) and 145.4 nm (“thick” fiber), and two different pore sizes, 0.22 μm (“dense” clot) and 1.1035 μm (“loose” clot). These values span a representative range of diameters and pore sizes from the literature^44^.

**Figure 1:**
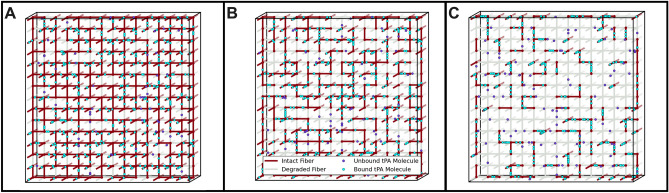
Establishing the model. A schematic of the model with intact fibers (red), degraded fibers (gray), unbound tPA (purple), and bound tPA (cyan) at 20% (**A**), 50% (**B**), and 80% (**C**) degradation.

Modeling scenarios were created to vary individual fibrin structure parameters in a way that cannot be done experimentally. Our previously established 3-dimensional (3D) macroscale model of a fibrin clot was modified to represent and isolate the different network and fiber conditions with tPA incorporated into the clot. Details about the model equations and development can be found in previous publications^25,31,39^. Briefly, the model is multiscale: a stochastic 2-dimensional (2D) microscale model of a single fiber cross-section (that contains detailed biochemistry of the fibrinolytic process) is run 50,000 times to generate data about tPA dwell times and single fiber lysis times that are used in a 3D macroscale model of a fibrin clot. The macroscale model clot is a 3D square lattice one fiber thick in the dimension coming out of the page (Fig. 1A–C), with periodic boundary conditions in that direction and reflecting boundary conditions in the other two dimensions. Each lattice edge represents a fibrin fiber. To mimic internal lysis, tPA molecules' initial locations are uniformly randomly distributed throughout the clot domain. The macroscale model tracks these tPA molecules in space and time as they diffuse, bind to and unbind from fibrin, and initiate fibrinolysis. When a tPA molecule binds to a fiber on the macroscale, we randomly sample from the microscale model empirical cumulative distribution functions (CDFs) to determine when that tPA molecule will unbind, and when (or if) that fiber degrades. When a fiber degrades, any tPA still bound to the fiber is considered to be on a large fibrin degradation product (FDP) that can diffuse if there is space around it but is unable to diffuse through pores. By “pores” we mean the initial distance between fibers in the clot; once lysis begins, pores will expand as fibers are removed from the simulation, and the FDPs are allowed to diffuse into those spaces. The tPA bound to these large FDPs kinetically unbinds at a random rate based on the physiological unbinding rate of tPA from fibrin (here taken to be k_off_ = 0.036 μM^−1^ s^−1^), and at that point is available for rebinding elsewhere. More specifically, when a tPA molecule binds to a fiber in the macroscale model, it is assigned both an unbinding time from the microscale model CDF and a uniformly distributed random number. If the random number is greater than the forced microscale unbinding probability (0.0852 for 72.7 nm-diameter fibers, 0.0729129 for 145.4 nm-diameter fibers), then tPA kinetically unbinds: after the length of time corresponding to unbinding time has passed, the tPA molecule unbinds from the fiber and is able to diffuse and rebind elsewhere. If the random number is less than the forced unbinding probability, then tPA was forced to unbind and we consider it to be bound to a small FDP that can diffuse freely throughout the clot and we assign it a waiting time equal to 1/k_off_. Once the tPA’s waiting time is reached, it kinetically unbinds from the small FDP and is available for rebinding. However, if the fiber to which tPA is bound degrades before the tPA molecule’s unbinding time is reached, then the tPA is considered to be bound to a large FDP and must wait until its previously-assigned unbinding time is reached before it can rebind elsewhere.

Figure 1 shows a representative scenario with tPA molecules (circles) incorporated into a clot with intact fibers (red). tPA molecules bind (cyan circles), which initiates the lytic cascade. Over time, the fibers breakdown (gray) and more of the tPA molecules are unbound (purple circles).

Table 2 describes the scenarios developed in the present study to analyze the effect of network structure and tPA concentration on internal lysis. Two diameter sizes were chosen: 72.7 nm for thin fibers (TN) and 145.4 nm for thick fibers (TK). Two pore sizes were chosen: 0.22 μm for dense networks (D) and 1.0135 μm for loose networks (L). Several amounts of tPA molecules were used to preserve the ratio of tPA to fibrin molecules (“Fixed Ratio”, FR) or the concentration of tPA (“Fixed Concentration”, FC). Fiber numbers were chosen to preserve the total amount of fibrin in all clots (5.6 × 10^7^ monomers), so clot volumes varied but total amount of fibrin was fixed. For example, a scenario with large pores (loose) has fewer fibers but a larger clot volume, while a clot with small pore size (dense) has more fibers and a smaller clot volume. The nomenclature is as follows: a clot with thin fibers and a loose network would be labeled as TN (thin)-L (loose), followed by the number of tPA molecules (i.e., 9350 molecules). The final label for this scenario is TN-L 9350, which represents a clot in which there are thin fibers, large pore size, and a relatively large number of tPA molecules. Alternatively, a clot could have a thick (TK) fiber and dense (D) network with 307 molecules of tPA (i.e., TK-D 307).

**Table 2:** Modeling scenarios. Italicized tPA: fibrin ratios denote a high ratio. Underlined tPA: fibrin ratios denote a low ratio. Bolded concentration of tPA denotes a fixed concentration. All decimal places are included for reproducibility.

Scenario name	Fiber diameter (nm) Thin (72.7 nm) vs Thick (145.4 nm)	Pore size (μm) Dense (0.22 μm) vs Loose (1.0135 μm)	# of tPA molecules	# of fibers in clot	tPA/ fibrin ratio (# tPA/ FB monomers) (× 10^–6^)	Concentration of tPA (#/μm^3^)
TN-L 9350	72.7	1.0135	9350	25,761	*164*	**0.922**
TN-L 307	72.7	1.0135	307	25,761	5.40	0.0303
TN-D 684	72.7	0.22	684	118,558	12.0	**0.923**
TN-D 9350	72.7	0.22	9350	118,558	*165*	12.6
TN-D 307	72.7	0.22	307	118,558	5.40	0.414
TK-L 3042	145.4	1.0135	3042	7453	53.6	**0.923**
TK-L 9350	145.4	1.0135	9350	7453	*165*	2.84
TK-L 307	145.4	1.0135	307	7453	5.41	0.0931
TK-D 307	145.4	0.22	307	34,208	5.43	**0.923**
TK-D 9350	145.4	0.22	9350	34,208	*165*	28.1

For all scenarios, ten independent simulations were run, with each simulation running until full clot degradation. The time step for the different scenarios depends on the diffusion coefficient of tPA and was chosen so that a molecule travels the distance between two fibers in one such interval. For dense clots, this is 1.613e-5 s while loose clots have a longer step of 3.424e-4 s. The degradation state of all fibers in the model is recorded at 10 s or 100 s intervals (determined manually to accommodate the total simulation time of the model). From this data we calculate the fraction of fibers that have degraded at a given point in time. The lysis lag time is defined as the first data record where at least 0.05 or 5% of the clot has degraded, with the time to 20, 50, and 80% lysis recorded similarly. Degradation rate for each simulation is determined by the slope of a least squares linear regression, fit to the data points in a specified interval. Intervals chosen were 20–50% lysis, 50–80% lysis, and 20–80% lysis. Molecule transit time was measured from the time a tPA molecule started moving (due to kinetically unbinding from an intact fiber or due to the breakup of the fiber to which it is bound) until the time the molecule bound to a new fiber. Pore size changes were measured by traversing evenly-spaced horizontal and vertical lines across the clot and recording the distance between intact fibers. To further observe the simulation results, we calculated the fold change for the time to 5% degradation, time to 50% degradation, and degradation rate for the thick/thin (to study diameter) or dense/loose (to study pore size) scenarios. Core model logic was implemented in custom Fortran code with Python used to transfer data files, input parameters, process results, and generate model state plots.

### Statistical analysis

All statistical analysis was performed using Prism 9.0. Outliers were identified and removed using a Grubbs' test with alpha of 0.05. Outliers (numbers in parentheses) were found using this technique for FR rate of formation (one), FC rate of formation (one), FC time to 50% clot (one), FR lysis lag time (one), FC lysis lag time (three, across all fibrinogen concentrations), FR time to 50% lysis (one), FR degradation rate (one), FC rate of degradation (one). Normality was confirmed using a D’Agostino and Pearson test for experiments; similar standard deviations were confirmed with an F test. Normality was assumed for modeling. One-way analysis of variance (ANOVA) with adjustments (Brown–Forsythe and Welch or Kruskal–Wallis) were used to compare fibrinogen concentrations. Individual tests are denoted in the figure legends. Two-way ANOVAs were used to compare fixed ratio (FR) and fixed concentration (FC). Alpha of 0.05 was used for all tests. Linear or decaying exponential fits were calculated for clotting/lysis times and degradation rates, respectively, for experimental conditions. Slopes and decay constants were recorded for analysis. Pearson correlation tests were performed to compare fibrinogen concentration and clotting/lysis parameters. Experiments were performed in biological and technical replicates.

All experimental data is presented as mean ± standard error of the mean; the supplement displays individual data points for transparency. Modeling data is presented as either mean ± standard deviation (SD) or individual data points with mean ± SD. Mean ± SD was used for modeling results when there were greater than 1000 values; raw data can be found on Zenodo^45^. Unless otherwise specified, *p < 0.05, **p < 0.01, ***p < 0.001, ****p < 0.0001.

### Ethical approval

The authors do not have any financial or personal relationships to disclose.

## Results

### Experimental results

#### A fixed concentration of tPA impairs fibrinolysis as the fibrinogen concentration increases, while fixed ratio of tPA molecules and fibrinogen molecules impairs lysis but to a lesser extent

**Figure 2:**
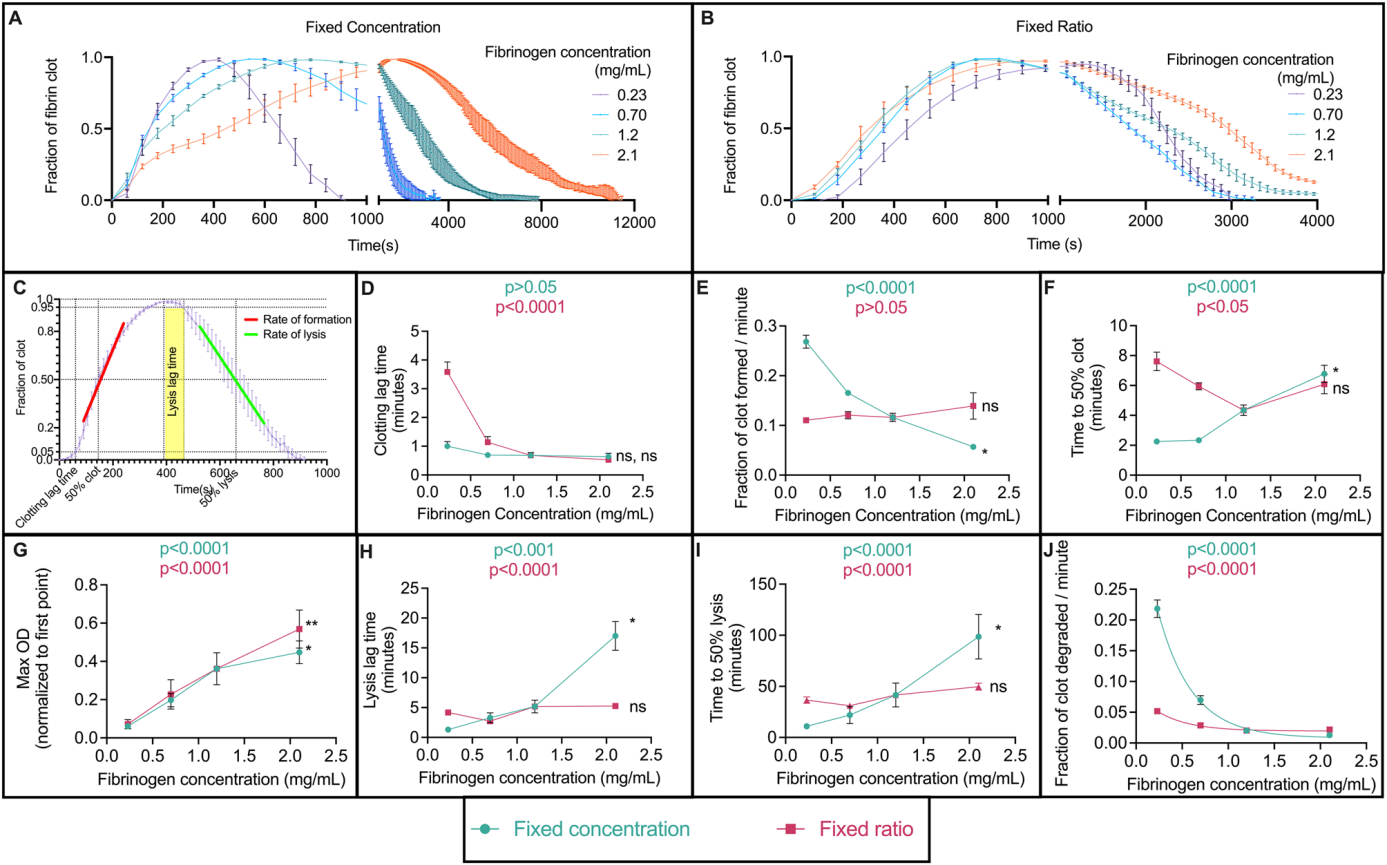
Clot formation and degradation of fibrin clots with varying fibrinogen concentrations. Fixed concentration of tPA (**A**) and ratio of tPA to fibrinogen (**B**) turbidity curves of four fibrinogen concentrations. A schematic diagram detailing six parameters measured to quantify clot formation and degradation (**C**). Clotting lag time (**D**), rate of formation (**E**), time to 50% clot formation (**F**), maximum optical density (**G**), lysis lag time (**H**), time to 50% lysis (**I**), and degradation rate (**J**) were measured and analyzed for all conditions. Symbols next to the curve denote the results from the correlation test (relationship between fibrinogen concentration (X axis) and clotting/lysis parameter (Y axis)): ns (no significance) *p* > 0.05, *p < 0.05, **p < 0.01. *p* values in each box denote the overall significance from the ANOVA Brown–Forsythe comparison test (comparing between fibrinogen concentrations within the tPA group). *Note* the *p* value is not related to the correlation coefficient. n = 9. Mean ± standard error of the mean.

First, we characterized clot formation and fibrinolysis when the same concentration of tPA was added to clots with differing fibrinogen compositions (and, therefore, different resulting structures). To mimic internal lysis, we performed experiments with a fixed concentration of tPA added uniformly throughout the volume prior to clotting. The fraction of clot was monitored to observe trends in formation and fibrinolysis (Fig. 2A,B). tPA concentration was optimized to ensure that all clots fully formed before degradation began (Supp. Figure 3A, B). We analyzed seven key parameters to quantify formation and degradation (Fig. 2C): clotting and lysis lag times, rates of formation and lysis, times to 50% clot and 50% lysis, and maximum optical density. Individual parameters and analysis can be found in the supplement (Supp. Figure 4).

Expectedly, for a fixed concentration of tPA (teal curves in Fig. 2D–J), the clotting lag time was similar for all fibrinogen concentrations (ns; Fig. 2D), because total amount of fibrinogen does not affect the time it takes for clotting to begin. Clots with lower concentrations of fibrinogen formed at a faster rate than clots with higher fibrinogen (*p* < 0.0001, Fig. 2E). At higher fibrinogen concentrations, there was more fibrinogen that needed to polymerize into fibers with the same amount of thrombin initiating the clotting. Thus, there was a significant delay in time to 50% clot formation (*p* < 0.0001, Fig. 2F). The maximum optical density increased as fibrinogen concentration increased (*p* < 0.0001, Fig. 2G), indicating denser clots. Unsurprisingly, these denser clots with more fibrinogen took longer to degrade; there was a delay in time to 5% (Fig. 2H, *p* < 0.001) and 50% (Fig. 2i, p < 0.0001) lysis as fibrinogen concentration increased. Likewise, the degradation rate, measured as the fraction of clot degraded per minute, decreased as the concentration of fibrinogen increased (*p* < 0.0001, Fig. 2J). There was a decaying exponential relationship (decay constant, k = 2.704) between degradation rate and fibrinogen concentration.

#### A fixed ratio of tPA molecules and fibrinogen molecules impairs lysis as fibrinogen concentration increases, but to a lesser extent than with a fixed concentration of tPA

It was previously suggested that a fixed ratio of tPA molecules to fibrin molecules would improve the efficacy in external fibrinolysis of dense clots compared to a fixed concentration of tPA^25^; here, we wanted to probe if the same is true for internal lysis. We performed experiments like the above, but we kept the ratio of tPA:fibrinogen molecules constant (FR, pink curves in Figs. 2D–J) as opposed to keeping the tPA concentration constant (FC) (Table 1). Thus, there was a higher concentration of tPA for a higher concentration of fibrinogen in order to maintain the ratio, as well as a lower tPA concentration for lower fibrinogen concentrations. Individual parameters and analysis can be found in the supplement (Supp. Figure 5).

Interestingly, there was a significant delay in clotting lag time for lower fibrinogen concentration when a fixed ratio of tPA to fibrin was used, which was not seen for a fixed concentration (*p* < 0.0001, Fig. 2D). This was unexpected but could be due to overall lower protein content in the clot during formation. However, there was no difference in rate of formation across fibrinogen concentrations (ns, Fig. 2E). There was minimal difference in the time it took to reach 50% clot formation (*p* < 0.05, Fig. 2F); the difference largely arose due to the lowest fibrinogen concentration taking longer than the rest of the conditions. This suggests an optimal fibrinogen concentration that minimizes time to 50% clot formation. Expectedly, the maximum optical density increases with comparable values to the fixed concentration case (*p* < 0.0001, Fig. 2G), since final clot structure should be mostly independent from amount of tPA present during formation.

**Figure 3:**
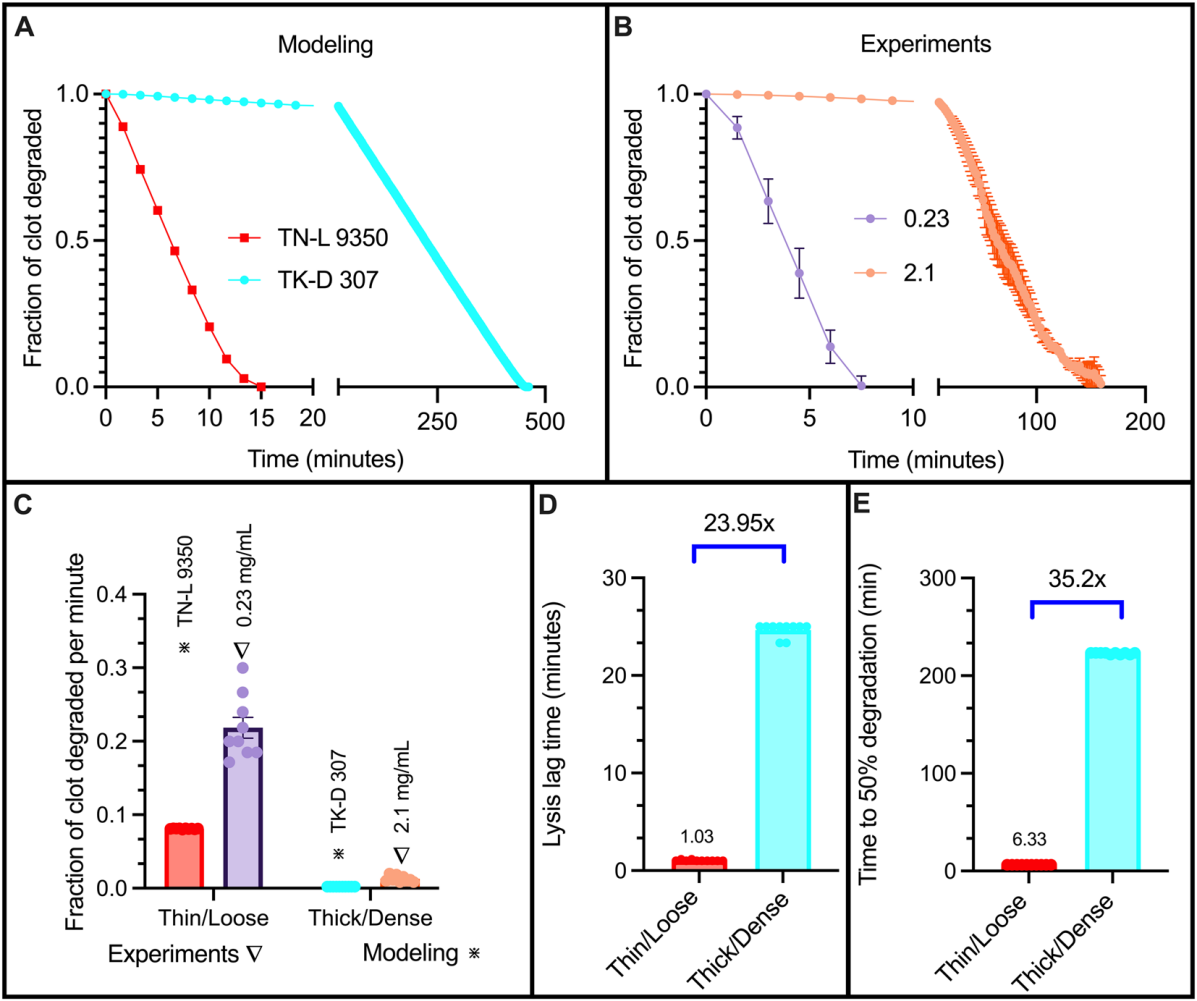
Validating the model with experimental comparison. Fraction of clot degraded as a function of time for modeling scenarios (**A**) and experiments (**B**). The absolute value of the degradation rate, measured as fraction of fibrin clot degraded per minute, was measured (**C**). The model lysis lag time (**D**) and delay (**E**) were recorded. Fold changes shown in D and E denote comparisons between the thick/dense and thin/loose scenarios.

The lysis lag time was significantly different for a fixed ratio of tPA to fibrinogen, but there was neither a trend nor a correlation between fibrinogen concentration and lysis lag time (*p* < 0.0001, Fig. 2H). Similarly, there was a significant difference in time to reach 50% lysis as the fibrinogen concentration increased (*p* < 0.0001, Fig. 2I), but without a trend or correlation. Interestingly, the 0.23 mg/mL fibrinogen sample had a longer lysis lag time and time to 50% lysis than the 0.70 mg/mL sample (Supp. Figure 5F). As fibrinogen concentration increased from 0.70 to 1.2 to 2.1 mg/mL, both delays in lysis continued to increase. The delay in time to 50% lysis for the 0.23 mg/mL sample was more similar to that of the sample with 1.2 mg/mL. Lastly, the degradation rate data for increasing fibrinogen concentration could be fit with a decaying exponential (*p* < 0.0001, Fig. 3J, k = 2.933) when there was a fixed ratio of tPA to fibrin.

We observed that the degradation delays and rates were subdued for FR versus FC. To study this behavior, we did a side-by-side comparison of fixed concentration and fixed ratio to observe the different behaviors (Supp. Figure 6A–C). We performed a two-way ANOVA with multiple comparisons to look at the effect on multiple levels. Interestingly, this revealed that the fixed ratio samples for lysis lag time, time to 50% lysis, and degradation rate were mostly statistically insignificant, while the opposite is true for fixed concentration. While statistical differences were observed when focusing on *either* FC or FR, when we performed analysis that studied both groups at once (a two-way ANOVA rather than a one-way ANOVA), the differences in clotting/lysis parameters seen between fibrinogen concentrations with a fixed ratio of tPA became insignificant compared to the differences seen for a fixed concentration of tPA.

We performed correlation tests to observe if there was a direct correlation between fibrinogen concentration and any of the formation or degradation parameters. For a fixed concentration of tPA, a correlation with fibrinogen concentration was identified for rate of clot formation (*p* < 0.05, Fig. 2E), time to 50% clot formation (*p* < 0.05, Fig. 2F), maximum optical density (*p* < 0.05, Fig. 2G), lysis lag time (*p* < 0.05, Fig. 2H), and time to 50% lysis (*p* < 0.05, Fig. 2I). For a fixed ratio of tPA to fibrinogen, the only correlation with fibrinogen concentration was identified for maximum optical density (*p* < 0.01, Fig. 2G).

### Modeling results

After confirmation that a fixed ratio of tPA molecules to fibrin molecules was more effective than a fixed concentration of tPA, we sought to identify the specific structural factors of the clot that drive this behavior. Experimentally, we and others identified that density, dictated by pore size, and diameter were the two key structural changes between clots with different fibrinogen levels (Supp. Figure 2)^30,31,46^. However, it is challenging experimentally to isolate these parameters as they are inherently linked; at a fixed thrombin concentration, higher fibrinogen concentrations result in clots with thicker fibers and smaller pores. We modified a previously established 3D-mathematical model of external fibrinolysis to simulate internal lysis of fibrin clots and to isolate the individual structural changes in a way that cannot be done experimentally. Individual raw data curves of the model can be found in the supplement (Supp. Figure 8). Modeling simulations were compared when only one structural parameter was different to isolate the dominant factor in reduced internal lysis of certain fibrin clots.

#### Validating the model

Before using the model to probe the mechanism of fibrinolytic resistance during internal lysis, we first validated that baseline conditions of the model matched experimental results. We chose two modeling scenarios that represented the baseline experimental conditions that had opposite structures: thin fibers/loose networks (0.23 mg/mL fibrinogen), and thick fibers/dense networks (2.1 mg/mL fibrinogen). The modeling scenarios varied fibrin fiber diameter (thin (TN) and thick (TK)) and fibrin network pore size (loose (L) and dense (D)). Hence, we compared the experimental low fibrinogen concentration results to a thin/loose model clot scenario (0.23 mg/mL and TN-L, respectively) and compared the experimental high fibrinogen concentration results to a thick/dense model clot scenario (2.1 mg/mL and TK-D, respectively). Unlike experiments, the model clots all contain the same amount of fibrin so that we can test the independent structural differences (pore size and diameter) that arise in the experimental clots. Using a fixed concentration of tPA (0.922 tPA molecules/μm^3^ for model and 40 ng/mL for experiments), we plotted the clot degradation curves (Fig. 3A,B). We quantified the degradation rate and observed that thin/loose clots degraded faster than thick/dense clots for both modeling and experiments (Fig. 3C). Next, we observed that the lysis lag time (Fig. 3D) and time to 50% lysis (Fig. 3E) were both delayed for thick/dense modeling scenarios, which is comparable to what was seen for experimental conditions (Fig. 3H,I). Side-by-side comparisons of lysis lag time and time to 50% degradation can be seen in the supplement (Supp. Figure 9A–C). Since we observed similar trends for all results, we confirmed that the model accurately represents the experimental conditions and can be used to further probe the mechanisms of internal lysis (Supp. Figure 9A–C). While we observed slight quantitative differences between modeling and experiments, the model’s qualitative trends matched experimental conditions which still allowed us to use the model to understand mechanisms.

#### A fixed ratio of tPA to fibrin reduces the resistance to lysis driven by reduced pore size

**Figure 4:**
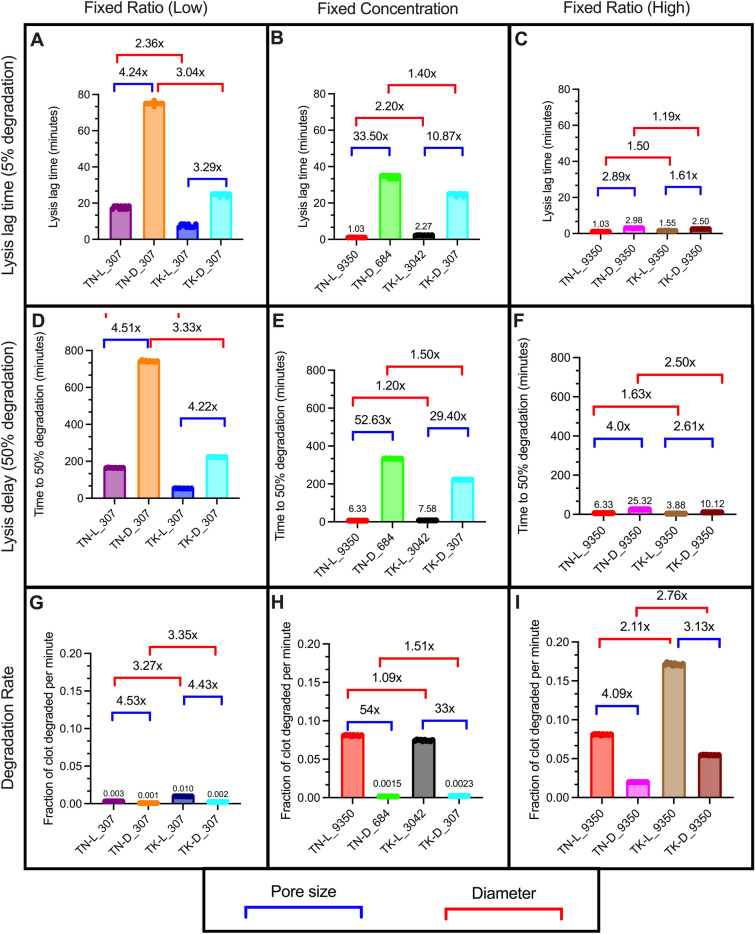
Comparing FC and FR with different network structures. Lysis lag time (**A**–**C**), lysis delay (**D**–**F**), and degradation rate (**G**–**I**) were recorded to compare the same concentration or ratio: “High” FR = 1.6e-4 tPA molecules per fibrin monomer (**A**, **D**, **G**), FC = 0.92 tPA molecules/μm^3^ (**B**, **E**, **H**), and “Low” FR = 5.4e-6 tPA molecules per fibrin monomer (**C**, **F**, **I**). Labels on the x-axis identify scenarios in which TN and TK refer to thin and thick diameters, respectively, L and D refer to loose and dense networks, respectively, and the numbers refer to the number of tPA molecules.

We analyzed the remaining model scenarios to study the role of fixed ratio (FR) and fixed concentration (FC) of tPA on lysis lag time, lysis delay, and degradation rate when structural parameters are changed. We focused on comparisons across changing pore size and diameter. For simplicity, we denote a fixed ratio of 5.40e-6 tPA molecules per fibrin monomer as the “Low” FR and 1.64e-4 as the “High” FR. First, we looked at the lysis lag time, or the time it took for 5% of the clot to degrade (Fig. 4A–C). For the most part, clots with fibers of thicker diameter had a shorter lysis lag times than their thinner counterparts, meaning that lysis started faster in these scenarios. The exceptions were loose network scenarios with a high FR or with a fixed concentration of tPA, in which the clots with thicker fibers were slower to initiate degradation than their thin diameter counterparts. As expected, denser networks consistently led to a longer lag time compared to their looser counterparts. Dense networks were slower to reach 50% lysis than loose networks, while networks composed of thick fibers reached 50% lysis faster than networks composed of thin fibers (Fig. 4D–F). Likewise, loose networks had a faster degradation rate than dense networks, and degradation rate was faster in clots with thick fibers compared to clots with thin fibers (Fig. 4G–I).

Overall, fold changes were largest when comparing pore size (rather than diameter); dense networks were more resistant to lysis compared to their loose counterparts. Furthermore, these observations were most exaggerated for a fixed concentration of tPA.

#### The extent of the effect of fibrin:tPA on fibrinolysis was structure-dependent

In the previous section, we focused on the role structure played when the concentration or ratio of tPA remained constant; here, we sought to understand how altering the amount of tPA influenced fibrinolysis when the clot structure was kept constant. We observed the impact of fixed tPA to fibrinogen ratio on the time it took for 5% of the clot to degrade (lysis lag time), the time it took for 50% of the clot to degrade (50% lysis time), and the degradation rate.

**Figure 5:**
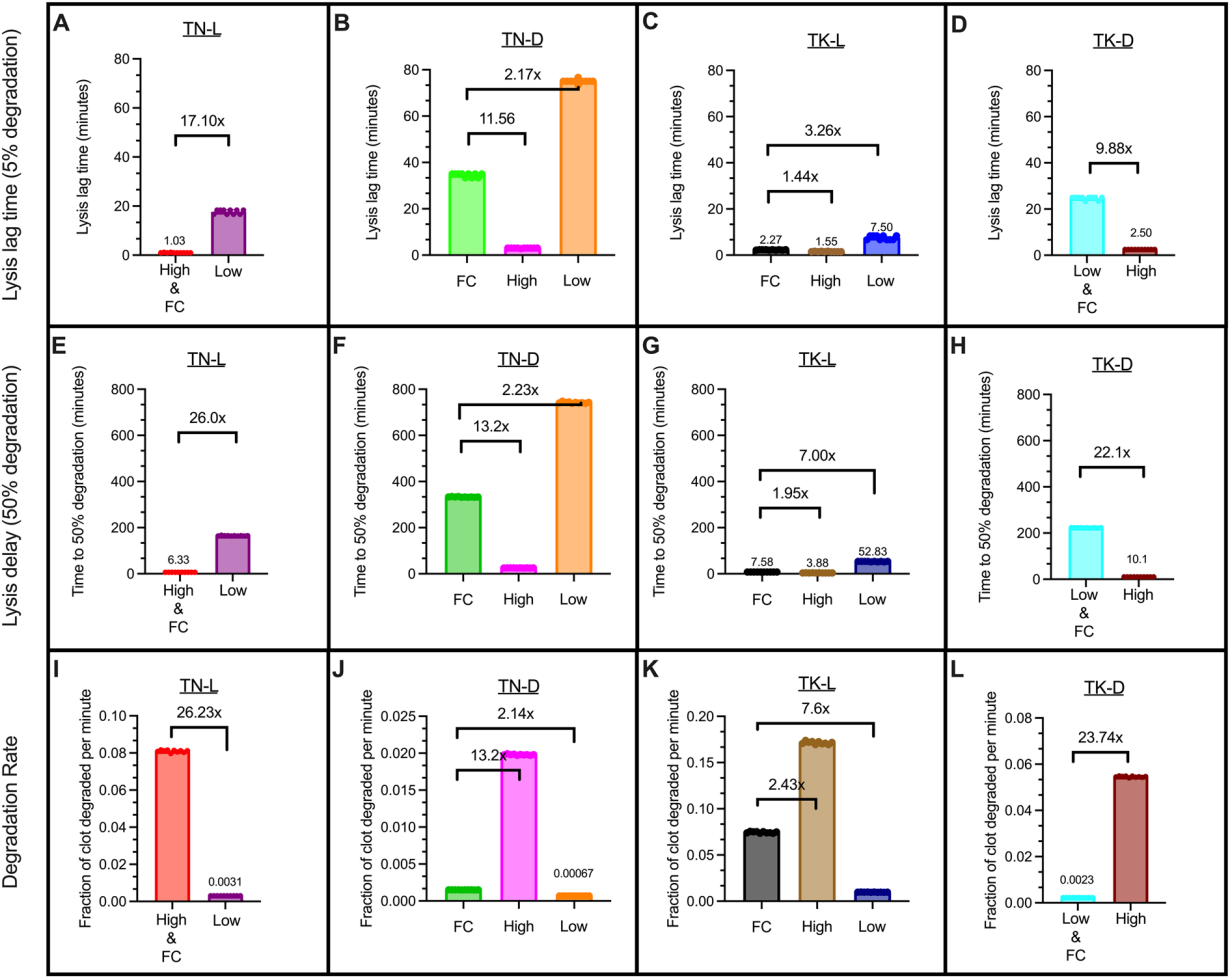
Comparing FC and FR within the same network structures. Lysis lag time (**A**–**D**), time to 50% lysis (**E**–**H**), and degradation rate (**I**–**L**) were recorded for four different clot structures: thin, loose TN-L (**A**, **E**, **I**); thin, dense TN-D (**B**, **F**, **J**); thick, loose TK-L (**C**, **G**, **K**); and thick, dense TK-D (**D**, **H**, **L**). “High” FR = 1.6e-4 tPA molecules per fibrin monomer, “Low” FR = 5.4e-6 tPA molecules per fibrin monomer, FC = 0.92 tPA molecules/μm^3^. Fold change is FC/FR. TN-L and TK-D scenarios only have two conditions because the amount of tPA for either the High (TN-L) or Low (TK-D) FR was the same as the FC. Labels on the x-axis identify scenarios in which TN and TK refer to thin and thick diameters, respectively, L and D refer to loose and dense networks, respectively, and the numbers refer to the number of tPA molecules.

A high FR resulted in a shorter lysis lag time for all structures compared to FC or to low FR (Fig. 5A–D), a shorter 50% lysis time (Fig. 5E–H), and a faster degradation rate (Fig. 5I–L). For all measured parameters, the largest fold changes between high FR and FC were for the TN-D and TK-D clots, which indicates that a small pore size (dense network) drove the observed differences. Furthermore, a low FR extended the lysis lag time (Fig. 5A–D), extended the 50% lysis time (Fig. 5E–H), and reduced the degradation rate (Fig. 5I–L) when compared to FC or high FR. This is unsurprising, as the low FR model scenarios contained the fewest number of tPA molecules (Table 2), and therefore were the slowest to both start and finish lysis. The biggest difference in FC vs high FR degradation rates was seen for the TK-D clot structure (Fig. 5L). The dense clot with thick fibers (TK-D) degraded very slowly with the fixed concentration of tPA used in the model. The next biggest difference in degradation rate was for the TN-D clot (Fig. 5J). We conclude that the dense network drove the difference in degradation rates between FC and high FR scenarios because when the impact of thick fibers was removed (but clot density remained the same), there was still a large increase in degradation rate compared to the FC scenario. Similarly, when comparing FC to low FR, we find that fiber density (rather than diameter) has the biggest influence on degradation rate; the biggest difference in degradation rate is in the TN-L clot scenario (Fig. 5I), followed by TK-L (Fig. 5K). Overall, we observed that the extent to which the ratio affected degradation was dependent on the diameter and pore size of the scenario.

#### Pore size expansion drives degradation

**Figure 6:**
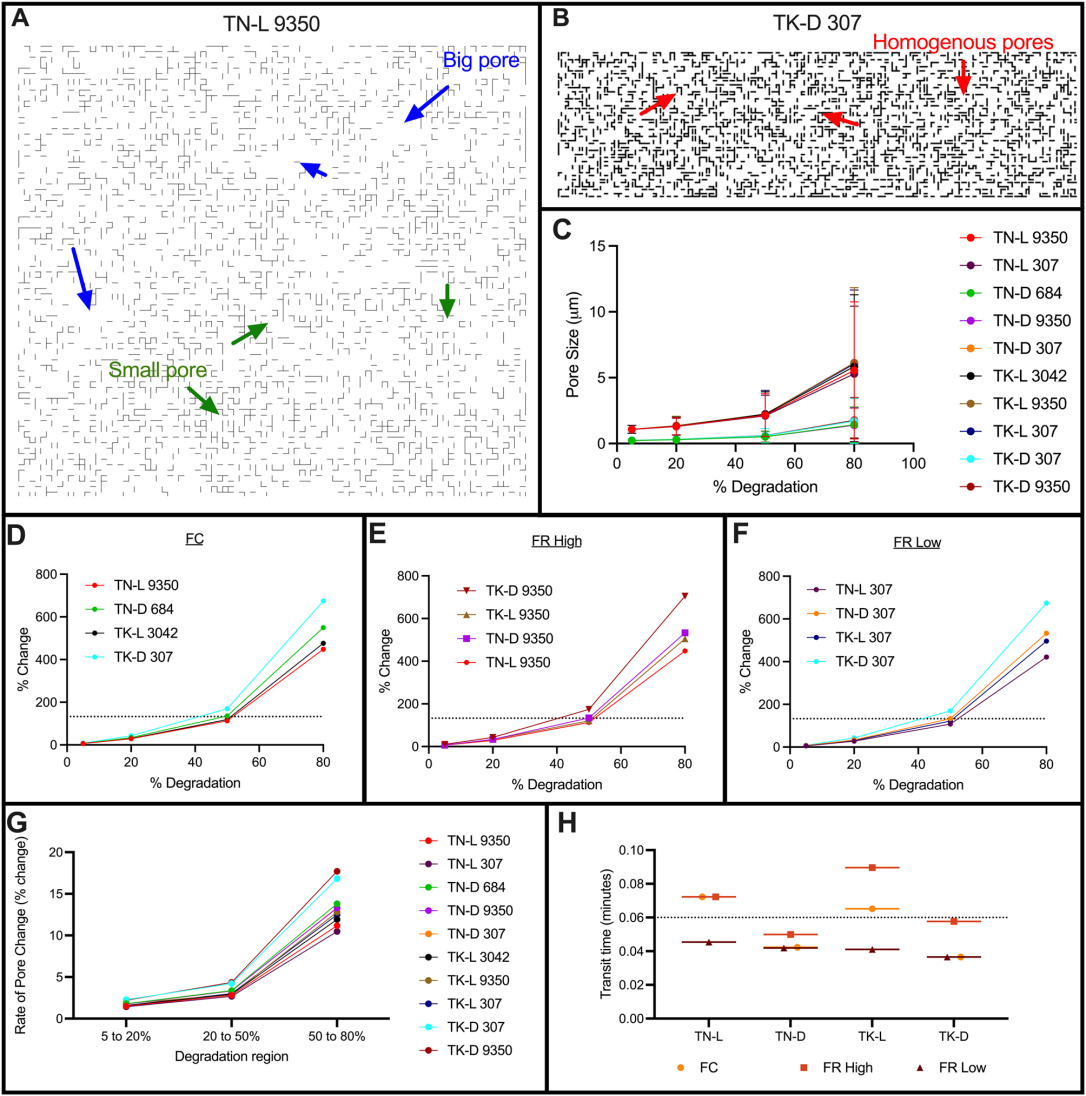
Pore expansion for modeling scenarios. Modeling schematics of TN-L 9350 (**A**), showing big (blue arrow) and small (green arrow) pores, and TK-D 307 (**B**) showing homogeneous pores (red arrow). Pore size (in microns) was measured as the clot degraded and presented as mean and standard deviation (**C**). Percent change over time was plotted for each modeling scenario with an FC (**D**), high FR (**E**), and low FR (**F**); dotted line indicates 132% change at 50% degradation. Rate of pore change (**G**) and tPA molecule transit times (**H**) were measured. The dotted line separates the shorter and longer transit times at 0.06 min. (**D**–**H**) are plotted as the mean.

**Figure 7:**
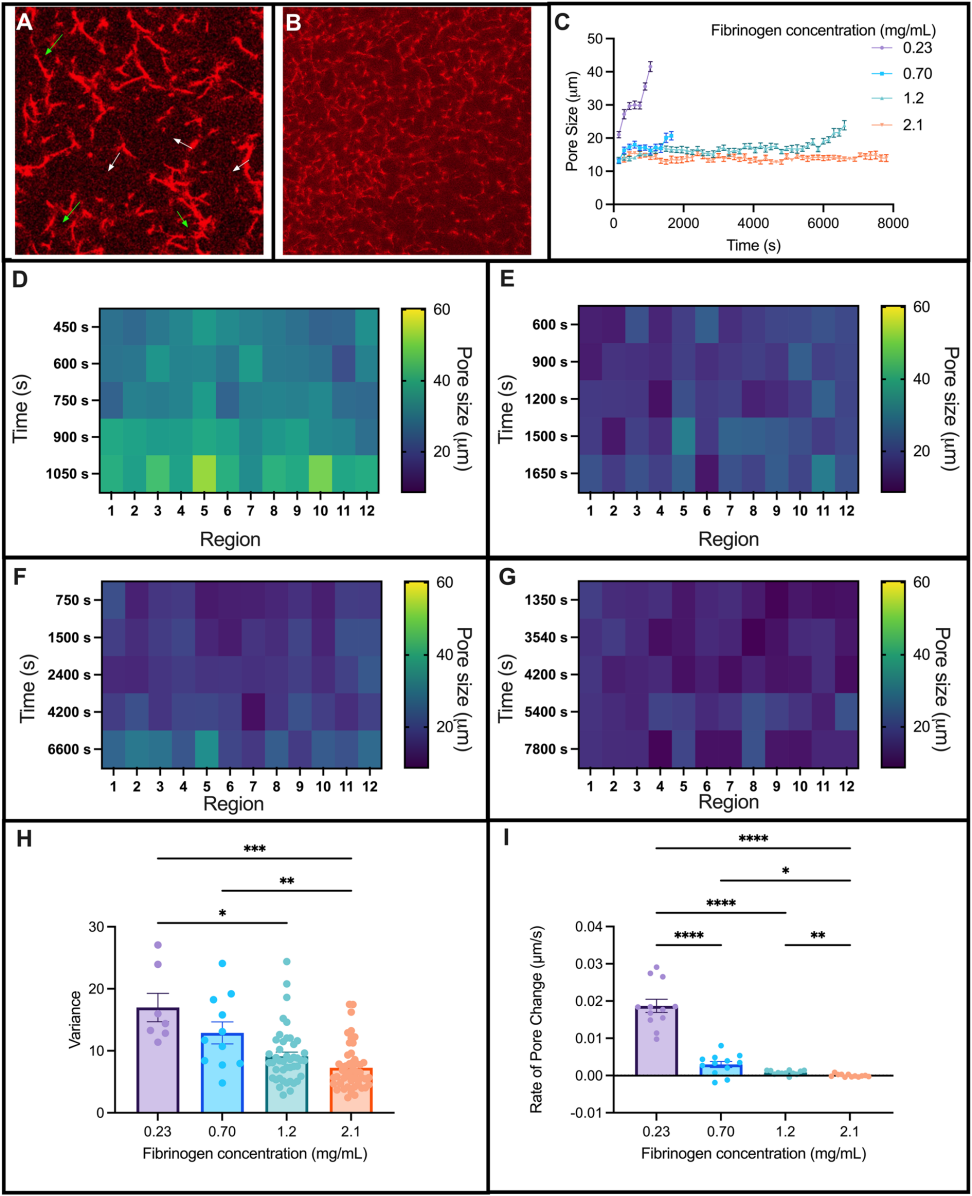
Pore expansion of the fibrin network during degradation. Confocal microscopy images of fibrin clots with heterogeneous (**A**) and homogeneous (**B**) pore expansion. Fibrinogen concentrations were 0.23 and 1.2 mg/mL, respectively. Pore size measured over time (**C**). Heat maps of pore sizes over time, measured in microns, for 0.23 (**D**), 0.70 (**E**), 1.2 (**F**), and 2.1 (**G**) mg/mL of fibrinogen at 12 different regions in the clot. Pore size variance at each timepoint (**H**) and rate of pore change, measured as microns per second (**I**). White arrow = large pores, green arrow = small pores.

Mathematical model generated schematics highlighted regions of degradation (growing pores) and regions of fibrin stability (intact fibers) (Fig. 6). As we validated the model using TN-L 9350 and TK-D 307 as the baseline conditions, we used them again here. From the model network plots, we observed that loose networks had heterogeneous pore expansion with both big and small pores at 80% degradation (TN-L 9350, Fig. 6A), while dense networks experienced more homogeneous pore expansion during breakdown (TK-D 307, Fig. 6B). We measured the pore size as the clot reached 5, 20, 50, and 80% degradation (Fig. 6C). Notably, the small pores of the dense networks remain small, even at 80% degradation; however, the pores of the loose network become much larger as degradation occurs. This suggests that loose networks have greater over overall changes in pore size compared to dense networks. Next, we observed that when pore size was normalized to the initial pore size, rapid pore expansion occurred at ~ 50% degradation and ~ 132% pore expansion for all scenarios (Fig. 6D–F). Likewise, the rate of pore change was similar for all conditions; the fastest rate for all scenarios occurred between 50 and 80% degradation (Fig. 7G). Surprisingly, the percent change for the dense networks were greater than those for the loose networks and showed a greater rate of percent pore change (Fig. 6D–G). However, the pore sizes of the initially dense networks remain small with a lower magnitude difference from 5 to 80% degradation compared to loose networks (loose: 4.692 µm, dense: 1.300 µm). Overall, the pore expansion of loose networks was much greater in magnitude compared to dense networks.

Lastly, we recorded the binding and unbinding times to measure the amount of time tPA molecules spend in transit between fibers. We identified that tPA molecules in loose networks spent more time in transit compared to molecules in dense networks (Fig. 6H, loose: 0.064 min vs dense: 0.044 min). Furthermore, a high FR increased the time in transit for both loose and dense clots while a low FR decreased the amount of time in transit. Interestingly, all structures had similar transit times when a low FR of tPA was present; the same was not true for a high FR. Moreover, the loose networks had tPA in transit longer when there was an FC or high FR, compared to all dense conditions. These observations indicated that pore expansion and transit time were structure dependent.

### Experimentally validating the modeling results: lower fibrinogen concentrations with a fixed concentration of tPA experience heterogeneous pore expansion

Since the model predicted that pore expansion drives fibrinolysis, we wanted to experimentally validate this observation using confocal microscopy (Supp. Figure 10). At low fibrinogen concentrations, we observed heterogeneous pore expansion across the clot in which there were regions of large pores and other regions of small pores (Fig. 7A). Clots with a higher concentration of fibrinogen exhibited a more homogeneous degradation in which fibers broke down uniformly with homogeneous pore expansion (Fig. 7B). To quantify pore expansion, we measured pores in regions across the clot over time until complete degradation (Fig. 7C).

Heat maps were generated to qualitatively observe the distribution of pore sizes for each fibrinogen concentration. We selected five time points to display in the heat maps: the time the fibers appeared, the time the clot was fully formed, the time the clot had fully degraded, and two intermediate timepoints. The lower fibrinogen concentrations (Fig. 7D,E) displayed larger distributions of colors, indicating a larger array of pore sizes. Higher fibrinogen concentrations (Fig. 7F,G) displayed smaller distributions of colors, indicating a smaller array of pore sizes. This was validated with the variance of pore sizes decreasing with increasing fibrinogen concentrations (Fig. 7H). Lastly, we measured the rate of pore change which revealed a significantly slower rate of change for higher fibrinogen concentrations (Fig. 7I). These results support what was seen with pore expansion of the modeling simulations with loose networks.

## Discussion

In this study we used a combination of mathematical modeling and laboratory experiments to explore the role of clot structure on internal fibrinolysis. We experimentally studied the dynamics of clot formation and lysis using kinetic tracking and isolated key structural parameters including pore size and diameter. Using a combined experimental and theoretical approach, we identified that network density drives degradation via pore size expansion during internal lysis. While it is known that clot structures are different in pathophysiological conditions, here we show that internal fibrinolysis is driven by pore expansion, which is network structure dependent. Limited or rapid pore expansion of the fibrin networks during degradation could explain why some people are more or less resistant to clot degradation, respectively. As we show here, this pore expansion mechanism of fibrinolysis is critically impacted by the relative fibrinogen concentration.

The conditions studied here include pooled human plasma with varying fibrinogen concentrations and a fixed concentration of tPA or fixed ratio of tPA to fibrin molecules, which resemble physiological conditions that need to be explored to better understand the success (or failure) of lysis in different individuals. Experimental results show that clots made with higher fibrinogen concentrations and a fixed tPA concentration have the greatest resistance to lysis (Fig. 2H–J). A fixed ratio of tPA molecules to fibrinogen monomers (rather than a fixed concentration of tPA), removes the correlation between fibrinogen concentration and clotting and lysis (Fig. 2D–F, H–J) but results in the same network structures (Fig. 2G). A decaying exponential fit for degradation rate is observed for both fixed tPA concentration and fixed tPA to fibrinogen ratio scenarios, with a dampened curve for the fixed ratio case (Fig. 2J). Overall, less significant changes and trends are observed for each parameter when a fixed ratio of tPA to fibrinogen is present (Supp. Figure 5) when compared to a fixed concentration (Supp. Figure 4). This suggests that a molecular ratio of tPA and fibrinogen drives clot degradation during internal lysis. Furthermore, this provides evidence that hemostasis is preserved when there is a careful balance of clotting factors and lytic enzymes. Monitoring these factors, in addition to others, and identifying the ratio could inform about *which* and *how much* therapeutics (i.e., supplemental fibrinogen or exogeneous tPA) could aid patients.

We use mathematical modeling to isolate the structural changes most responsible for the reduction of internal lysis in clots with high fibrinogen concentration. We adjust our preexisting model of external lysis to account for internal lysis by distributing tPA molecules randomly inside the preformed fibrin clot^31,39^. Modeling simulations reveal that pore size (rather than fiber diameter) plays the dominant role in the effectiveness of internal lysis (Figs. 4, 5, 6). Since disease conditions (i.e., fibrinogen concentration) play a role in resistance to degradation, future work can focus on pore size variations found under these conditions. A tailored research target (i.e., pore size) will aid in the fast advancement of understanding internal lysis and optimizing therapeutics to improve external lysis. Dense networks consistently lead to greater resistance to degradation with longer lysis lag times (Fig. 3A–C), extended 50% lysis times (Fig. 3D–F), and slower degradation rates (Fig. 3G–I). The overall fold differences observed for a fixed concentration of tPA (Fig. 3B,E,H) were larger than those with a fixed ratio of tPA and fibrin for all lysis measurements. This is not surprising as we propose that the fixed ratio preserves the ratio that is necessary for degradation. More importantly, the model identifies rapid or limited pore expansion for loose or dense fibrin networks, respectively (Fig. 6).

We show that pore size (rather than diameter) is the structural feature that dictates the extent of fibrinolytic resistance due to pore expansion. We propose that at high concentrations, tPA molecules cluster together leading to degradation and pore expansion in that region. When this behavior occurs in loose networks with little fibrin(ogen) and excess tPA, rapid pore expansion leads to excess degradation, which could explain why patients with such conditions (low fibrinogen + high tPA) are at risk of bleeding. In looser networks, tPA molecules travel a relatively shorter distance between binding events, clumping together at nearby fibers (Supp. Figure 12). Contrastingly, in dense networks with high fibrinogen and too little tPA, there is limited pore expansion and a subsequent resistance to degradation. In these scenarios, tPA molecules travel farther through the clot between binding events (Supp. Figure 12), resulting in less clumping and more homogeneous, but slower, degradation. This resembles hypofibrinolytic states in which patients are at risk of thrombosis. However, when there is an optimal concentration of tPA with respect to the concentration of fibrinogen, there is an ideal fibrin surface area for tPA molecules to bind for effective degradation and coordinated pore expansion. This scenario balances the clotting and lytic factors to achieve hemostasis.

The work presented here establishes a new understanding of the structural changes that occur during fibrinolysis. There are several future steps that could advance this work. All experiments and modeling were done in the absence of blood flow, which is a reasonable assumption for a completely occlusive thrombus. Future work could investigate the effect of flow on internal lysis. Also, since experiments were performed with diluted plasma, the range of fibrinogen concentrations was limited. Future work could explore higher fibrinogen concentrations (> 4 mg/mL,^47^) to imitate hypercoagulable states. Similarly, plasma was diluted with buffer to preserve the ratio between clotting factors and fibrinogen in the plasma, resembling situations that could result in lower fibrinogen concentrations^48^, as well as following similar protocols to clinical coagulation tests^49^. This future work could instead dilute the plasma with fibrinogen-depleted plasma to keep other clotting factor concentrations fixed. While we verified that the delayed lysis in clots with higher fibrinogen concentration was not an effect of the higher plasminogen activator inhibitor-1 (PAI-1) concentration in those samples (Supp. Figure 13), future work with both the model and experiments could consider inhibitors, such as PAI-1, and other coagulation factors, such as FXIII. Finally, experiments could be repeated with fixed fibrinogen concentrations, but varying thrombin concentrations, which would also lead to clots of different structures. By comparing those results to the results presented here, we could better understand how the total amount of fibrinogen (not just clot structure) affects internal lysis. Nonetheless, the present study altered the fibrin network structure with both modeling and experiments to delineate the role of tPA molecules and identify pore expansion as the primary structural driver of internal fibrinolysis rate. This study creates a foundation to study internal fibrinolysis with distinct clot conditions and an updated mathematical model.

## Conclusion

The present study sought to understand the biochemical and structural regulation of internal fibrinolysis. We showed that fibrinolysis is dependent on the fibrin network structure and the ratio of tPA to fibrin molecules. Moreover, our findings indicate that the pore size of the fibrin network dictates the resistance to internal fibrinolysis. For large network pore sizes seen in clots formed from lower fibrinogen concentrations, tPA creates a wave of degradation expanding outward from its initial location, resulting in local pore size expansion. We propose that with larger pore sizes, tPA clusters on fibers; with a larger distance to travel between fibers, tPA is more likely to rebind quickly to the fiber it is currently near, resulting in a ripple effect of degradation. Contrastingly, in clots with smaller pore sizes, like those formed with higher fibrinogen concentrations, the tPA molecules have more surface area of fibers to cluster around which results in a more homogeneous breakdown of the clot.

This work establishes a foundation to understand how tPA molecules present inside the fibrin network work in concert with the clot structure to influence internal lysis. Knowledge gained here can be used to understand what promotes internal fibrinolysis to optimize therapeutics to mimic effective internal lysis. Further, our studies encourage a novel approach for future diagnostics to factor in a patient’s endogenous tPA:fibrin composition. Such criteria may help preemptively identify patients inherently at risk for impaired fibrinolysis. Future work will incorporate internal and external tPA to better imitate physiological conditions.

### Supplementary Information

Supplementary Information.

## Data Availability

The modeling data that support the findings of this study are openly available in Zenodo at http://doi.org/10.5281/zenodo.8115180, reference number 8115180. The experimental datasets used and/or analysed during the current study are available from the corresponding author on reasonable request.
