# Supplementary material for: Internal fibrinolysis of fibrin clots is driven by pore expansion
Source: Sci Rep. 2024 Feb 1;14:2623. doi: 10.1038/s41598-024-52844-4 (PMC10830469; doi:10.1038/s41598-024-52844-4)
Supplement: Supplementary file 1 — Supplementary Information. [file 41598_2024_52844_MOESM1_ESM.docx]

### Supplement

Internal fibrinolysis of fibrin clots is driven by pore expansion

Rebecca A Risman^a^, Bradley Paynter^b^, Victoria Percoco^a^, Mitali Shroff^c^, Brittany E Bannish^b^, Valerie Tutwiler^a,*^

1. Rutgers University, Department of Biomedical Engineering
2. University of Central Oklahoma, Department of Mathematics and Statistics
3. Rutgers University, Department of Cell Biology and Neuroscience

**Supp. Figure 1.** Dilution with buffer vs depleted plasma

**Supp. Figure 2.** Structural analysis with confocal microscopy

**Supp. Figure 3:** Raw experimental data and lysis curves

**Supp. Figure 4:** Fixed concentration of tPA

**Supp. Figure 5:** Fixed ratio of tPA:fibrin molecules

**Supp. Figure 6:** Fixed concentration and fixed ratio

**Supp. Figure 7:** Two-part lysis

**Supp. Figure 8:** Raw modeling scenario curves

**Supp. Figure 9:** Establishing the model.

**Supp. Figure 10:** Confocal images of timelapses

**Supp. Figure 11:** How pore size was measured for experiments

**Supp. Figure 12:** Normalized tPA diffusion distance

**Supp. Figure 13:** Role of PAI-1 on fibrinolysis.

1. Dilution with buffer vs depleted plasma

To confirm results were a consequence of changing fibrinogen concentration, we conducted an experiment comparing plasma diluted with buffer vs fibrinogen-depleted plasma. The fibrinogen was depleted from the same stock of 2.4 mg/mL fibrinogen plasma using batroxobin (1). For this subset study, we used a turbidity assay to look at the 1.2 mg/mL sample (50% dilution) with buffer or fibrinogen-depleted plasma (Supp. Figure 1A below). First, we saw an insignificant difference in rate of formation (Supp. Figure 1B). Next, we saw an insignificant difference in max OD (Supp. Figure 1C), which indicates minimal changes in clot structure. Lastly, we saw insignificant differences in the time to 50% lysis (Supp. Figure 1D) and degradation rate (Supp. Figure 1E).

**Supp. Figure 1. Buffer vs. fib(rinogen)-dep(leted) plasma.** A) Turbidity assay tracking clot formation and lysis with plasma diluted with buffer or fibrinogen-depleted plasma. B) Rate of formation, C) max optical density, D) time to 50% lysis, and E) degradation rate can be measured from turbidity curves. ns p>0.05. N=2.

1. Increasing fibrinogen concentration results in denser networks with thicker fibers

To study the structure of the clot, we performed confocal microscopy and scanning electron microscopy (SEM). For confocal microscopy, clots were formed with the same conditions as described in turbidity methods with the addition of 1% by plasma volume AlexaFluor 488 nm (FITC) labeled fibrinogen. 100 µL of each sample was pipetted into a 96-well black bottom glass plate before clotting occurred. Samples were allowed to clot at room temperature for at least 30 minutes. Images were captured on a Zeiss 780 confocal microscope with 40x, water objective, magnification and numerical aperture of 1.2. Pore size and density were measured using FIJI. Images were converted to binary with a black background and white fibers and density was characterized as the percent area of the white region.

In order to determine the influence of fibrin network structure on fibrinolysis, we assessed pore size and % area covered by fibers. With increasing fibrinogen concentrations, there was an increase in % area covered by fibers and a decrease in pore size. This is consistent with what has been reported previously (Risman 2022). The pores of the fibrin network are the region where tPA can diffuse through the network.


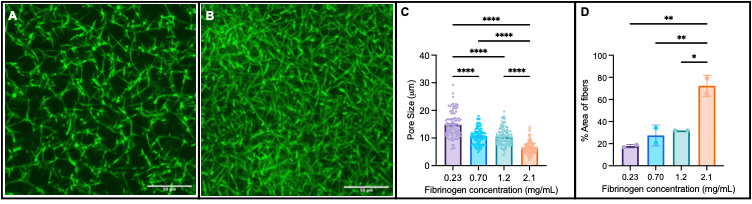


**Supp. Figure 2. Fibrin network structure.** Confocal microscopy of fibrin networks with 0.23 (A) and 2.1 (B) mg/mL fibrinogen concentrations. Pore size (C) and percent area of fibers (D) were measured for each condition (One-way ANOVAs). Scale bar is 50 microns.

1. Raw data curves

Fibrinogen concentrations were diluted from plasma with a fibrinogen concentration of 2.9 mg/mL. Four concentrations were used to observe trends. The fixed tPA concentration was chosen to ensure clotting was not impaired. The fixed ratio was calculated based on the ratio of tPA to fibrin for the 1.2 mg/mL fixed concentration case. Lysis-only curves are shown to compare to the model.


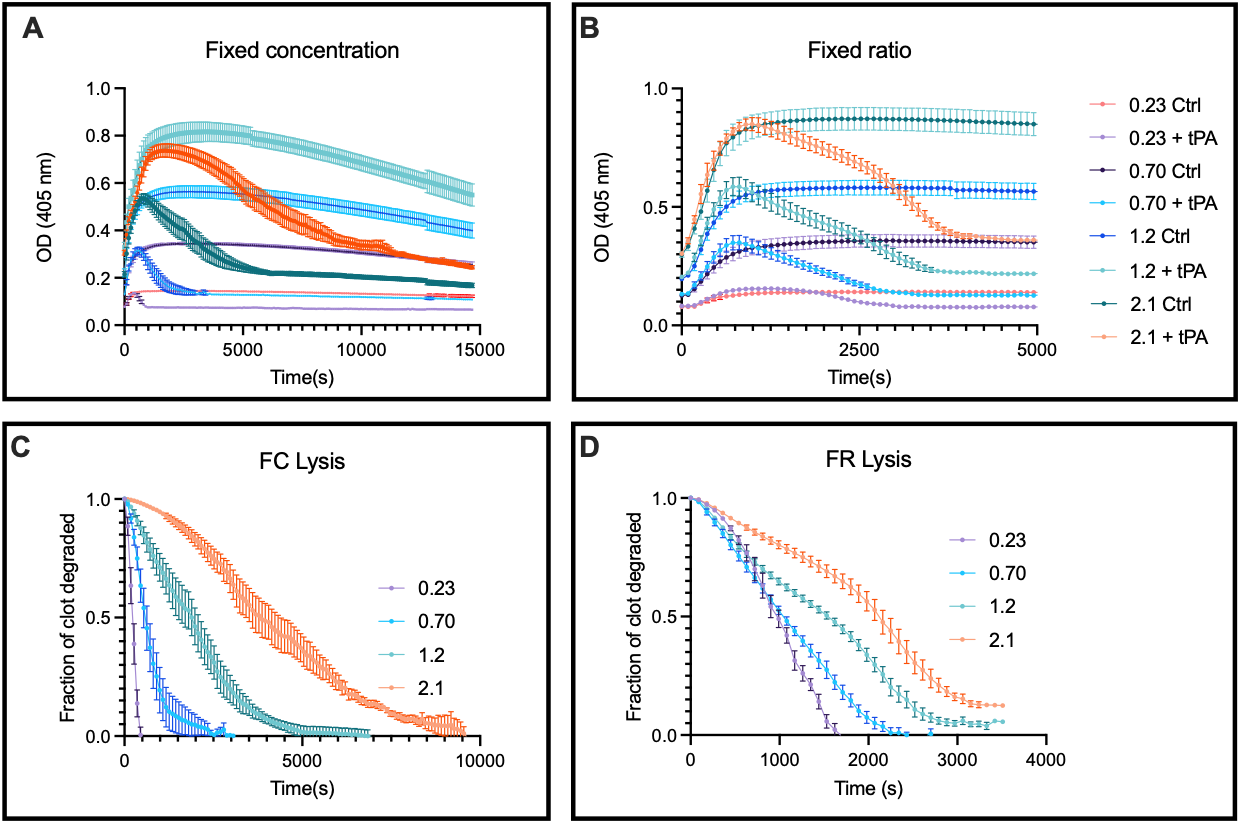


**Supp. Figure 3. Raw experimental data and lysis curves.** Raw data curves for FC (A) and FR (B). Lysis-only curves for FC (C) and FR (D). N=3, n=9. Mean ± standard error of the mean.

1. Individual data for turbidity

The main text highlights the overall trends and comparisons of clot formation and fibrinolysis for fixed concentration (FC) and fixed ratio (FR). Here, we separate FC and FR to observe statistical significance between fibrinogen concentrations for each tPA condition.


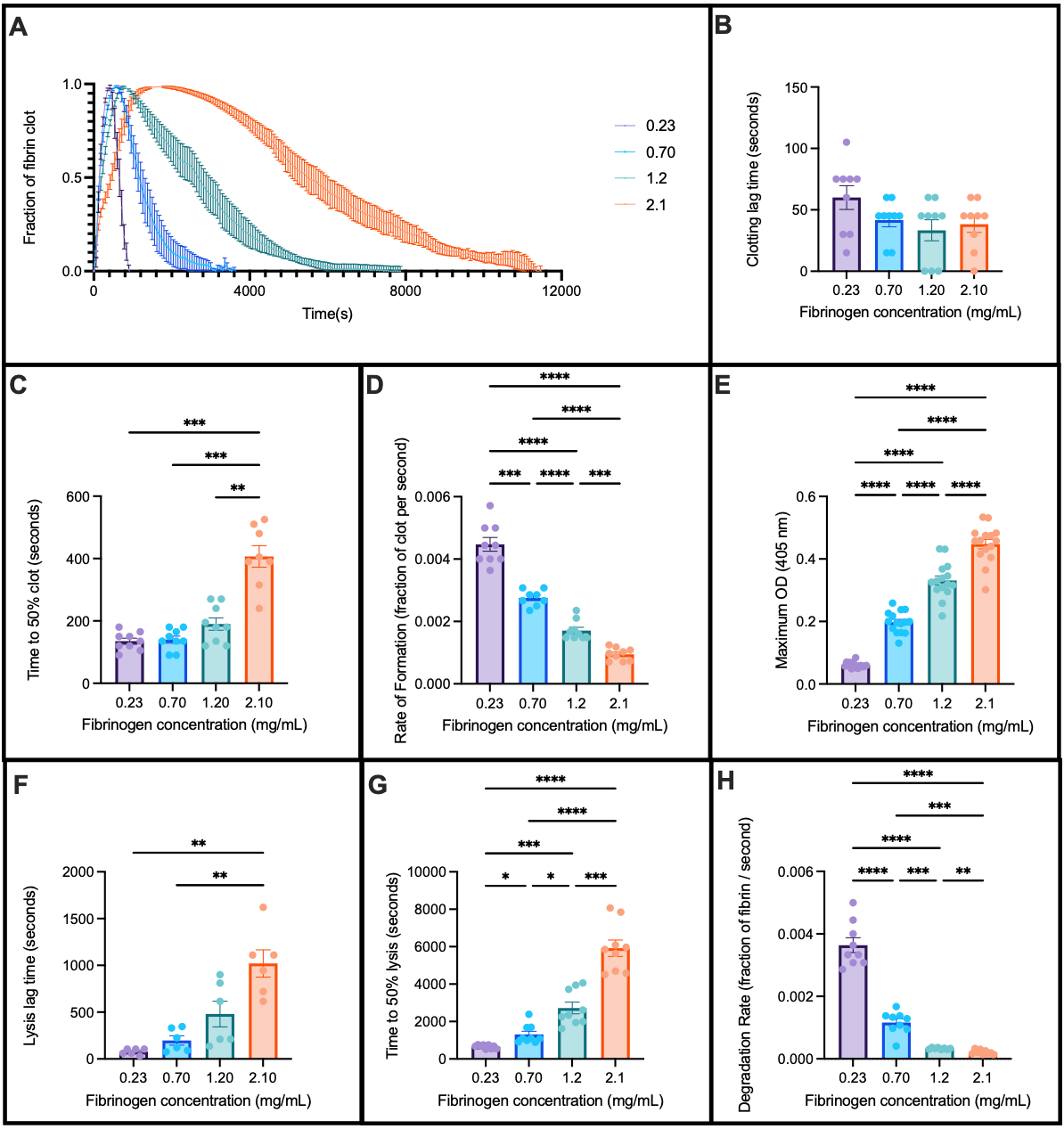


**Supp. Figure 4: Fixed concentration of tPA.** Clot formation and degradation of the clot measured as a fraction of fibrin clot (A). Clot formation was quantified with clotting lag time (B), time to 50% clot (C), rate of formation (D), and maximum optical density (E). Clot degradation was quantified with lysis lag time (F), time to 50% lysis (G), and degradation rate (H). Comparisons with p>0.05 are not displayed. * p<0.05, ** p<0.01, *** p<0.001, **** p<0.0001. N=3, n=9.


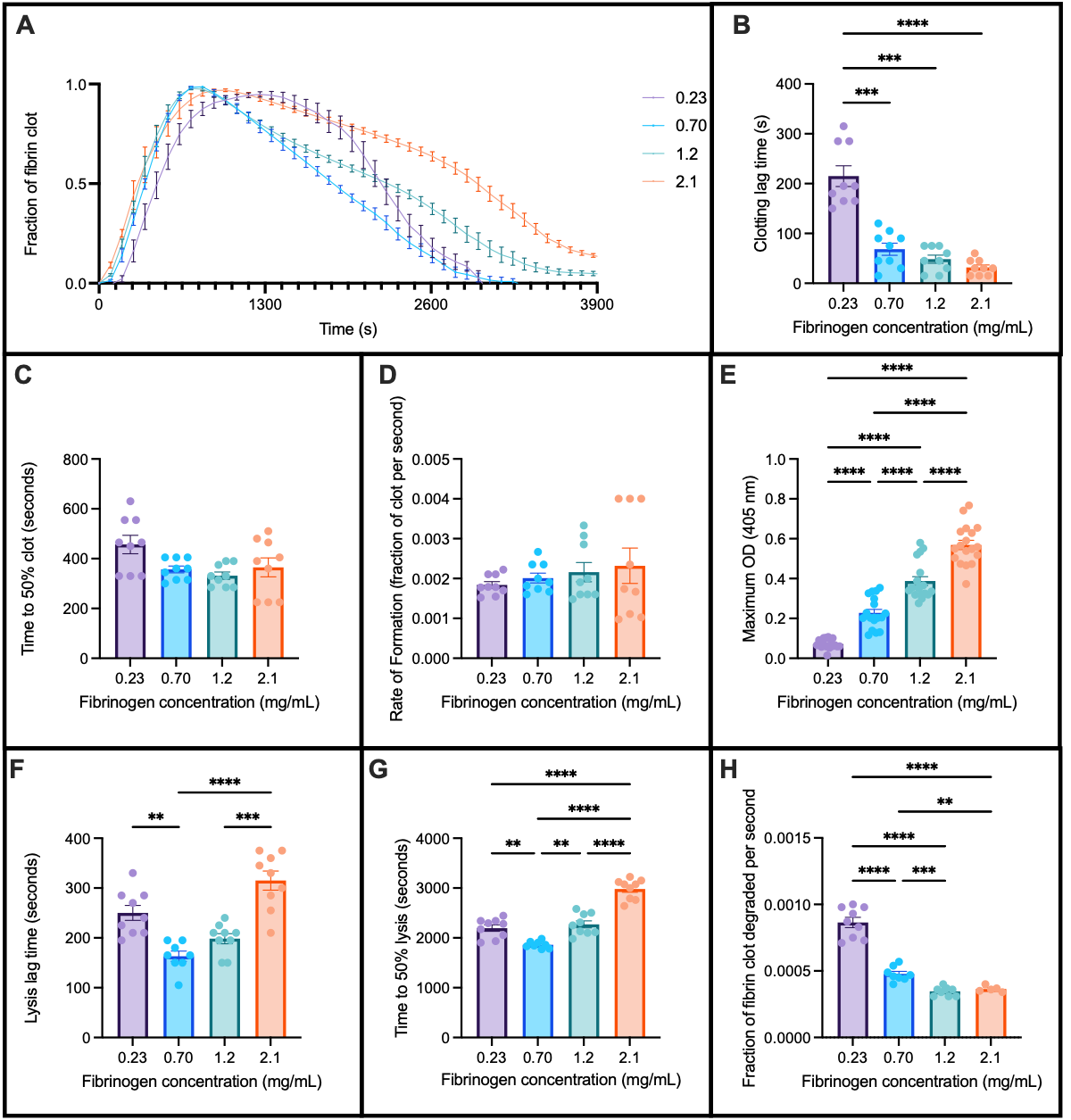


**Supp. Figure 5: Fixed ratio of tPA.** Clot formation and degradation of the clot measured as a fraction of fibrin clot (A). Clot formation was quantified with clotting lag time (B), time to 50% clot (C), rate of formation (D), and maximum optical density (E). Clot degradation was quantified with lysis lag time (F), time to 50% lysis (G), and degradation rate (H). Comparisons with p>0.05 are not displayed. * p<0.05, ** p<0.01, *** p<0.001, **** p<0.0001. N=3, n=9.

**Supp. Figure 6:** **Side-by-side of fixed concentration and fixed ratio.** Fixed concentration and fixed ratio with a two-way ANOVA of lysis lag time (A), time to 50% lysis (B), and degradation rate (C).

1. Two-part lysis

For some fixed ratio conditions, we observed a two-part fibrinolytic process in which one rate accounts for lysis up to 50% clot degradation, while another rate is seen from 50% to complete degradation (Supp. Figure 6A, B). Interestingly, the trend for which rate was faster was fibrinogen-concentration-dependent. For fibrin clots with fibrinogen concentration of 0.23 mg/mL with a fixed ratio of tPA, the clot broke down faster after 50% lysis (p<0.001, Supp. Figure 6B). At 0.70 mg/mL and 1.2 mg/mL fibrinogen, the rates before and after 50% lysis were not significantly different (ns, Supp. Figure 6B). The trend flipped at 2.1 mg/mL fibrinogen, in which the degradation started out faster then slowed down after 50% of the clot was degraded (p<0.01, Supp. Figure 6B). This suggests a structurally driven transition in which lysis at higher fibrinogen concentrations starts slower then speeds up over time, and vice versa for lower fibrinogen concentrations. Notably, these differing degradation rates are only seen in FR (not FC) experiments. This could allude to the mechanism in which FR was able to optimize the degradation; a dual affect with the structure may be occurring earlier and later during lysis. This will be probed further in the future.

Previous work in the field has acknowledged diameter thickening during lysis while the present study observed pore expansion (2). For this reason, we propose that initially, the thinner fibers of the clots with lower fibrinogen concentration are more readily lysed while the thicker fibers of the higher fibrinogen concentration clots lyse slower, but only when there is a fixed ratio of tPA molecules. Later, pore expansion drives degradation in clots with lower fibrinogen concentration, while playing a more muted role in clots with higher fibrinogen concentration.


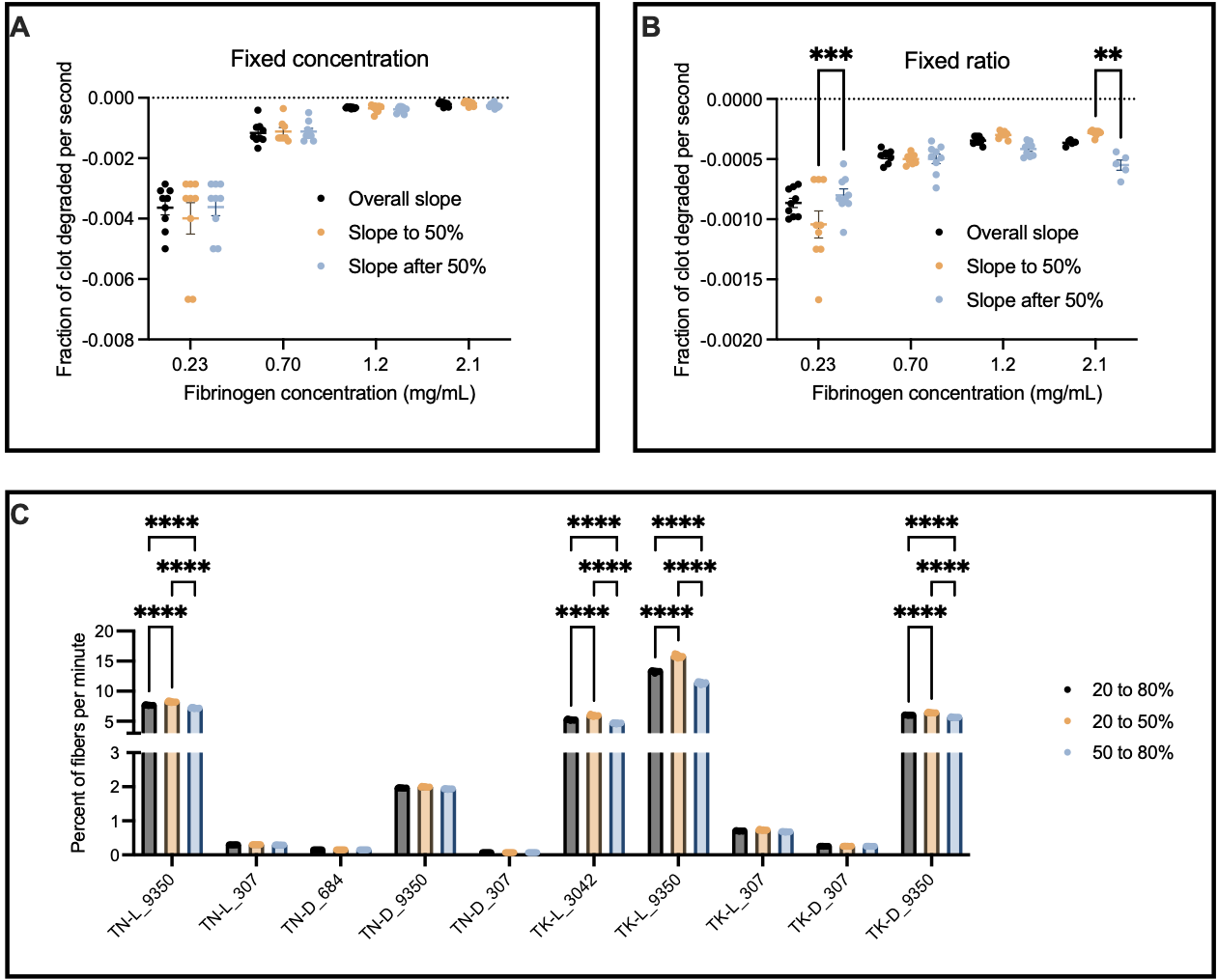


**Supp. Figure 7: Two-part lysis.** Overall degradation rate, rate before 50% lysis, and rate after 50% lysis for fixed concentration of tPA (A) and fixed ratio of tPA:fibrin (B).

1. Modeling raw data and establishment


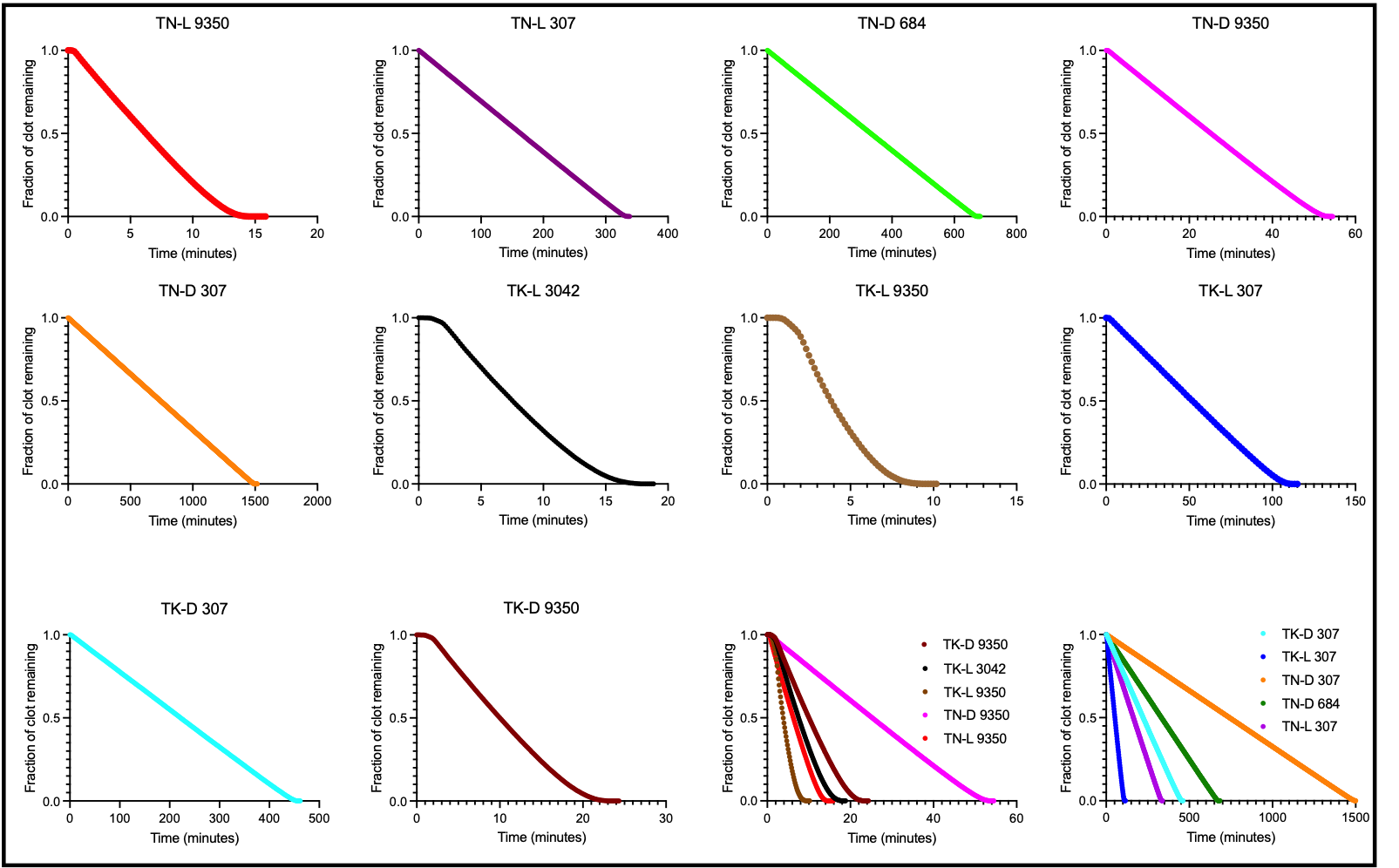


**Supp. Figure 8: Modeling scenario curves.** Raw data curves of each modeling scenario as fraction of clot remaining over time.


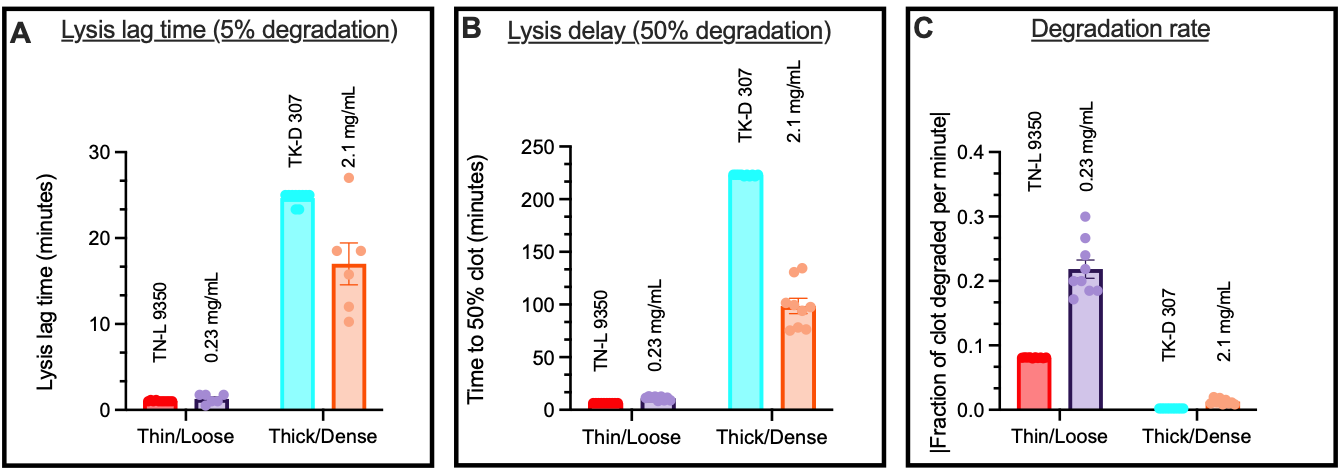


**Supp. Figure 9: Establishing the model (continued).** Side-by-side comparison of modeling and experiments with thin/loose vs thick/dense with lysis lag time (5% degradation) (A), lysis delay (50% degradation) (B), and degradation rate (C).

7. Kinetic monitoring of network degradation with confocal microscopy


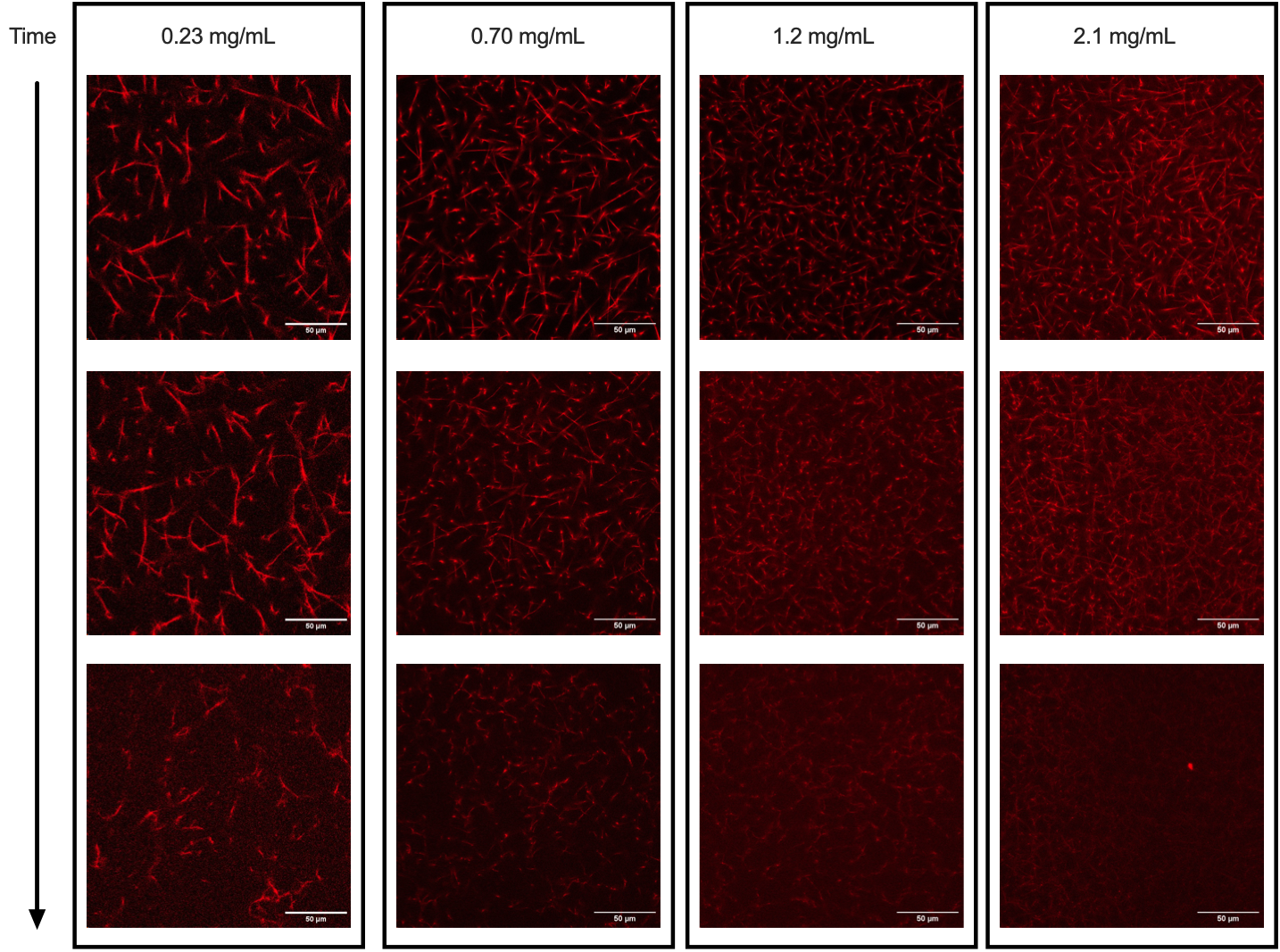


**Supp. Figure 10: Confocal images of timelapses.** Scale bar is 50 microns.


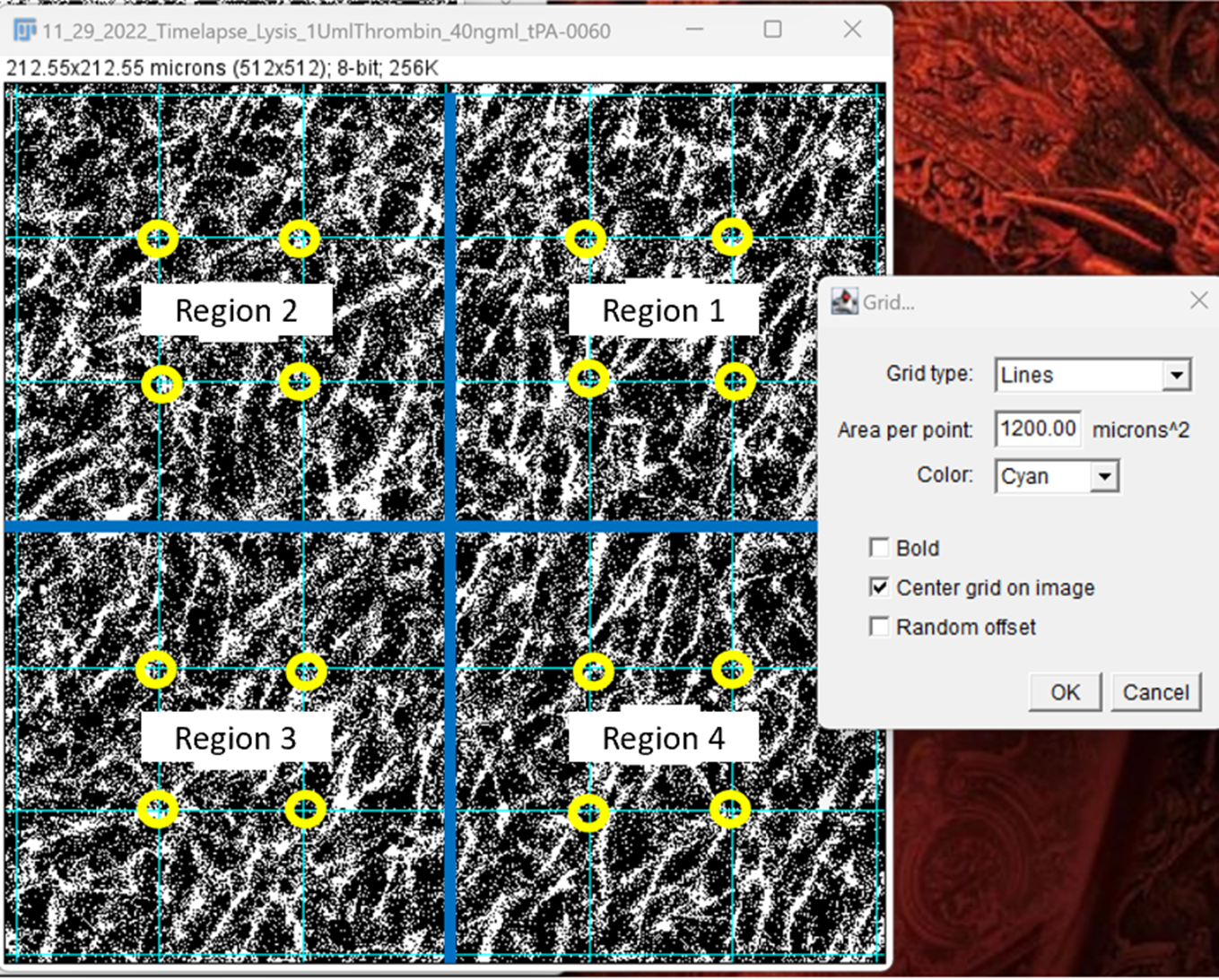


**Supp. Figure 11: How pore size was measured**

Pore size measurements were collected using the FIJI software. A standard method for measuring pore sizes was utilized to compare fibrin fiber networks created with different fibrinogen concentrations. Each time lapse was converted to its binary form, split into individual images (of 30 second intervals), and overlaid with a grid of 1200 micron squared. Each image was further split into four quadrants. Pore size measurements were taken every 5 and 10 timelapse frames. Four pores were measured in each quadrant. Measurement locations corresponded to the grid intersections to minimize bias in selecting pores. Standard deviation and variance of regional pore sizes across regions were recorded for each time point. Rate of pore expansion was found by fitting a line to the recorded pore measurements from beginning of formation until degradation for each clot region. The slopes were averaged for each condition.

8. Normalized tPA diffusion distance

**Supp. Figure 12: Normalized tPA diffusion distance. Mean distance diffused by tPA between two consecutive binding events (for molecules that started moving during the previous interval of degradation [10(i-1)% to 10i%]), normalized by the mean pore size in the clot at the end of the degradation interval (10i%, i=2...8). The symbols for loose clots are open circles with dotted lines; dense clots are denoted by solid circles with straight lines.**

In Supp. Figure 11 we plot the mean distance traveled by a tPA molecule, normalized by the network pore size, at different percentages of clot degradation. All curves decrease as more of the clot is degraded because the pore sizes become bigger, and hence the denominator in the (distance traveled)/(pore size) calculation becomes larger. This figure shows that mean distance traveled by tPA relative to pore size is greater in dense clots than in loose clots, supporting our conclusion that tPA is more likely in loose clots to bind nearby to where it unbound, and more likely in dense clots to spread out.

9. Role of PAI-1


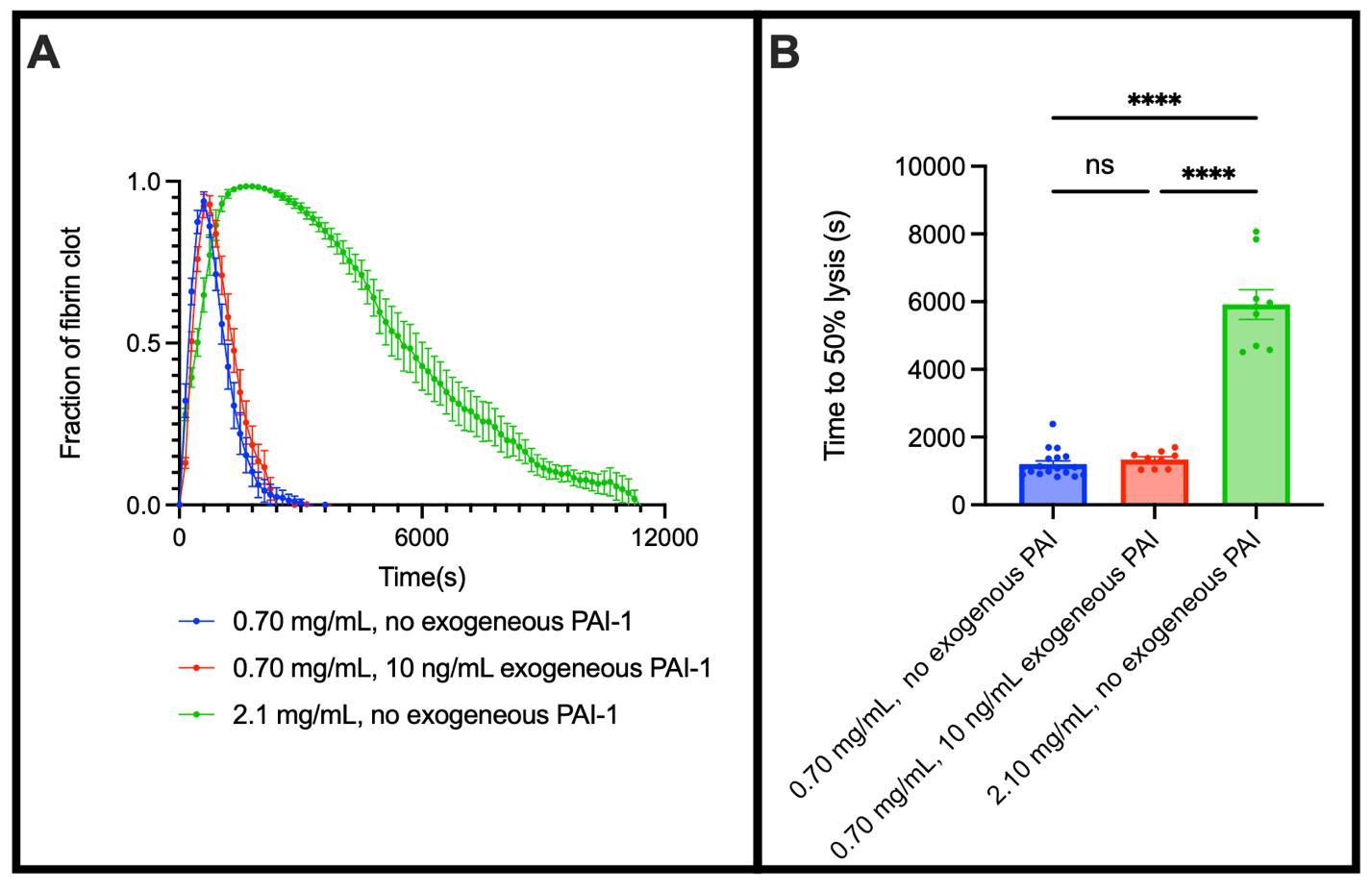


**Supp. Figure 13: Role of PAI-1 on fibrinolysis. A) Turbidity experiment with varying PAI-1 or fibrinogen concentrations. B) Time to 50% lysis. ns p>0.05, **** p<0.0001.**

To specifically address the effect of PAI-1, we conducted an experiment where we either added exogenous PAI-1 to a diluted sample or increased the fibrinogen concentration without adding exogenous PAI-1 (Supp. Figure 11A). All samples had 40 ng/mL of tPA. The control clot had 0.70 mg/mL fibrinogen (achieved through dilution), so the PAI-1 was also diluted. The second sample had 0.70 mg/mL fibrinogen (achieved through dilution) plus 10 ng/mL of exogenous PAI-1 (Sigma A8111-25UG) added. The addition of PAI-1 had an insignificant effect on lysis time (Supp. Figure 11B). The last sample had 2.1 mg/mL of fibrinogen, which is in the physiological range, with no exogenous PAI-1. There is a significant delay in lysis of this clot (Supp. Figure 11B). Changing the PAI-1 concentration in two samples with the same fibrinogen concentration (0.70 mg/ml fibrinogen) makes a 1.1x difference in time to 50% lysis; changing the fibrinogen concentration from 0.70 to, 2.1 mg/ml while not adding exogenous PAI-1 makes a 4.9x difference in time to 50% lysis. This suggests that the delay in lysis for samples with lower dilutions (higher fibrinogen concentrations) was not the effect of PAI-1.

(1) Garyfallogiannis K, Ramanujam RK, Litvinov RI, Yu T, Nagaswami C, Bassani JL, et al. Fracture toughness of fibrin gels as a function of protein volume fraction: Mechanical origins. Acta Biomaterialia 2023;159:49-62.

(2) Lynch SR, Laverty SM, Bannish BE, Hudson NE. Microscale structural changes of individual fibrin fibers during fibrinolysis. Acta biomaterialia 2022;141:114-122.
